# Reliability of the Biomechanical Assessment of the Sagittal Lumbar Spine and Pelvis on Radiographs Used in Clinical Practice: A Systematic Review of the Literature

**DOI:** 10.3390/jcm13164650

**Published:** 2024-08-08

**Authors:** Joseph W. Betz, Douglas F. Lightstone, Paul A. Oakley, Jason W. Haas, Ibrahim M. Moustafa, Deed E. Harrison

**Affiliations:** 1 Private Practice, Boise, ID 83709, USA; drjoebetz@gmail.com; 2 Institute for Spinal Health and Performance, Cumming, GA 30041, USA; douglas.lightstone@gmail.com; 3 Kinesiology and Health Science, York University, Toronto, ON M3J 1P3, Canada; docoakley.icc@gmail.com; 4 Chiropractic Biophysics NonProfit, Inc., Eagle, ID 83616, USA; drjason@idealspine.com; 5 Department of Physiotherapy, College of Health Sciences, University of Sharjah, Sharjah 27272, United Arab Emirates; iabuamr@sharjah.ac.ae; 6 Neuromusculoskeletal Rehabilitation Research Group, RIMHS—Research Institute of Medical and Health Sciences, University of Sharjah, Sharjah 27272, United Arab Emirates

**Keywords:** spine, sagittal view, lumbar, lordosis, pelvic morphology, radiography, X-ray, reliability, chiropractic, subluxation, systematic review of literature

## Abstract

**Background:** Biomechanical analysis of the sagittal alignment of the lumbar spine and pelvis on radiographs is common in clinical practices including chiropractic, physical therapy, scoliosis-related thoraco-lumbo-sacral orthosis (TLSO) management, orthopedics, and neurosurgery. Of specific interest is the assessment of pelvic morphology and the relationship between angle of pelvic incidence, sacral slope, and lumbar lordosis to pain, disability, and clinical treatment of spine conditions. The current state of the literature on the reliability of common methods quantifying these parameters on radiographs is limited. **Methods**: The objective of this systematic review is to identify and review the available studies on the reliability of different methods of biomechanical analysis of sagittal lumbo-pelvic parameters used in clinical practice. Our review followed the recommendations of the preferred reporting items for systematic reviews and meta-analyses (PRISMA). The design of this systematic review was registered with PROSPERO (CRD42023379873). **Results:** The search strategy yielded a total of 2387 articles. A total of 1539 articles were screened after deduplication and exclusion by automation tools, leaving 473 full-text articles that were retrieved. After exclusion, 64 articles met the inclusion criteria. The preponderance of the evidence showed good to excellent reliability for biomechanical assessment of sagittal lumbo-pelvic spine alignment. **Conclusions:** The results of this systematic review of the literature show that sagittal radiographic analysis of spinal biomechanics and alignment of the human lumbo-pelvic spine is a reliable tool for aiding diagnosis and management in clinical settings.

## 1. Introduction

The evaluation, origination, and treatment of spinal conditions are some of the most pressing research interest areas in today’s healthcare due to the general crisis of suffering globally and the expected increase in spine disorders in those under 40 years of age [1]. A major basis underlying the understanding and treatment of spine disorders is the reliability of sagittal radiographic analysis of spinal biomechanical alignment of the human lumbo-pelvic spine to assist clinicians in diagnosis and determination of the cause of their patient’s pain and disability [2,3]. It is given that if spine conditions can be evaluated reliably and proper diagnosis can be made this would contribute to appropriate treatment options for spine-related patient care [2,3,4,5]. In the end, reducing the global burden of musculoskeletal disorders is a desirable clinical outcome globally and reliable and valid assessment procedures are mandatory [1]. 

Contemporary surgical techniques for lumbar spine fusion surgery are dependent on the analysis of biomechanical parameters of the spine and pelvis from imaging techniques [3,4]. Variation in lumbo-pelvic morphology and lumbar sagittal alignment has been shown to predispose to adjacent segment degeneration following lumbar spine fusion surgery [2], as well as having correlation with long-term outcomes after instrumented fusion [5]. Recent studies show a predictive validity of different spinopelvic parameters on outcomes with scoliosis orthoses such as thoraco-lumbar and lumbo-sacral and combination thoraco-lumbo-sacral orthoses (TLSO) [6,7,8]. A mismatch between the pelvic morphology and lumbar lordosis as measured on radiographs has been shown to predict pain in a group with chronic low back pain compared to a matched control group [9] and the mismatch of pelvic morphology vs. lumbar lordosis has been linked to poor surgical outcomes as well [10]. Furthermore, contemporary spine rehabilitation techniques utilize radiographic imaging of the spine for the analysis of segmental and regional misalignment, as well as global sagittal balance, as a foundational component of their system of patient management with promising results [11]. A recent systematic review and meta-analysis demonstrated a strong correlation between the loss of lumbar lordosis and the presence of low back pain [12]. Another systematic review of prospective cohort studies showed reduced lumbar lordosis as one factor associated with an increased risk of developing LBP over a 12-month period [13].

Good examiner reliability of spinal displacement analysis on radiographs is essential when these analysis methods are used in clinical practice [9,10,11]. While there are many different methods of sagittal lumbar spine and pelvic analyses [2,3,5,9,10,11], our search found no previously reported systematic review on the reliability of lumbo-pelvic radiographic analytical methods. This is in contrast to the anterior-posterior lumbo-pelvic radiographic view in which a recent systematic literature review identified that radiographic measurements were the ‘gold standard’ for reliability and validity assessments of leg length inequality [14]. Despite sagittal lumbo-pelvic radiographic measurement methods being commonly used across spine care disciplines, debate exists as to the reliability and validity of these procedures and the relationship of sagittal spine alignment with health, including spinal pain, is often challenged [15,16,17,18,19]. We identified one recent non-systematic, rapid review performed on the reliability and validity of all regions of spine alignment and the relationship between spine alignment parameters and health [18]. This rapid review was sanctioned by the Chiropractic College of British Columbia and, based on its questionable findings, resulted in limiting the role of radiography utilization in chiropractic practice in British Columbia [20]. It is important to note that this article by Corso et al. [18] was critically appraised and found to be biased and overly restricted in their search strategy, including limiting the inclusion criteria to papers exclusively where the assessment or intervention was only performed by chiropractic authors [21] while ignoring any article performed by other healthcare providers.

Accordingly, there appears to be a considerable and rather urgent need to assess the literature on the reliability of lumbo-pelvic radiographic analytical methods for the assessment of spine displacements [21]. The aim of this systematic review of literature (SROL) is to review the scientific literature on the reliability of sagittal radiographic analysis of spinal biomechanics and alignment of the human lumbo-pelvic spine. This SROL will present the extent and quality of data on the topic and should contribute to the understanding of lumbo-pelvic radiographic evaluation, diagnosis, and management of spinal conditions. The hypothesis of the current SROL, and in contrast to some of the literature on this topic [15,16,17,18,19], is that a variety of radiographic lumbo-pelvic measurement methods will have good to excellent intra- and inter-examiner reliability; we base this hypothesis on the generalized popularity of these methods and the number of decades they have been utilized across spine disciplines.

## 2. Materials and Methods

The design of this systematic review was registered with PROSPERO (CRD42023379873). To optimize the reporting within this systematic review, we followed the preferred reporting items for systematic reviews and meta-analyses (PRISMA) 2020 statement and flow diagram [22]. Although PRISMA 2020 was designed for systematic reviews of studies on the effects of health interventions, many items are applicable to reviewing other types of studies [22]. The use of PRISMA in this review ensures clarity in why this review was completed, what the investigators performed, and what was found as a result. The most recent [22] 27-item checklist was utilized at all points of the investigation, interpretation, presentation, and reportage of the findings.

### 2.1. Search Strategy

**Figure 1:**
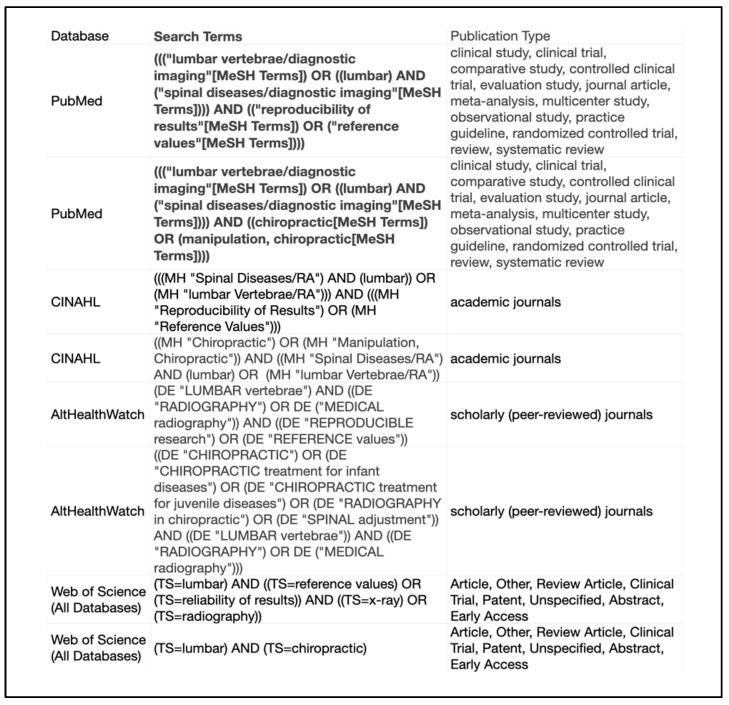
Search strategy.

In accordance with the PRISMA checklist, our study utilized a patient/population, intervention, comparisons, and outcomes (PICO) alternate. The PICO alternate design for the search strategy followed the peer-review of electronic search strategies (PRESS) checklist [23]. This included using an independent librarian with a Master of Librarian and Information Science to design the search strategy with the peer-review of a second independent library sciences specialist. Databases searched included PubMed, Cumulative Index to Nursing and Allied Health Literature (CINAHL), Alt Health Watch, and Web of Science (all databases) from the date of database inception through 4 January 2023. Search strategies for each database are provided in Figure 1.

### 2.2. Inclusion Criteria

Inclusion criteria maintained that studies were published and available in English, performed on humans only, and used radiography of the sagittal lumbar spine and/or pelvis on either asymptomatic controls or participants with defined spinal conditions and were reliability studies of biomechanical measures of the sagittal lumbo-pelvic spine.

### 2.3. Exclusion Criteria

Studies were excluded if they were animal investigations, geometric modeling studies, cadaver studies, and studies performed with a mannequin “phantom” for X-ray investigation. Studies were excluded if they utilized non-radiographic advanced imaging methods such as magnetic resonance imaging (MRI), ultrasound (US), thermal imaging, surface contour only, etc. All studies that did not perform a reliability analysis of lateral lumbo-pelvic biomechanical parameters were excluded from the final manuscripts reviewed.

### 2.4. Study Selection

The initial strategy provided 2387 titles and abstracts that met the inclusion and exclusion criteria. Automation tools were further able to screen studies according to the inclusion and exclusion criteria from terms as well as for duplicates between different databases and narrowed the search to 1539 articles. Titles and abstracts for the 1539 articles were screened independently by two authors according to the inclusion and exclusion criteria. Discrepancies between the reviewers were settled by a third reviewer and a consensus was achieved. This resulted in 473 articles meeting all inclusion criteria. Review of the full-text articles to determine eligibility was conducted by two reviewers independent of one another. A third reviewer collected the results from the first two reviewers and discrepancies for inclusion were made by consensus. No automation tools were used by the reviewers to screen the 473 articles. This resulted in 64 articles in the data collection stage and study bias analysis.

### 2.5. Data and Study Characteristics Collection

Data were collected by authors J.W.B. and D.F.L. and spreadsheets were created in Microsoft Excel. The two reviewers independently collected the data and evaluated defined characteristics from each included study. As full texts and abstracts were reviewed, the investigators compared each study to the inclusion and exclusion criteria and used assessments for bias. Discrepancies in data and study characteristics collection were adjudicated by a third reviewer (J.W.H.) and consensus was achieved.

The information collected from each study included participant data (number, age, sex, etc.), number of examiners, details on the repeat analysis of the reliability study, type of imaging used, methods of biomechanical analysis, statistical analysis of inter- and/or intra-rater reliability, and results of the statistical analysis.

### 2.6. Study Bias and Quality Assessment

The quality appraisal for reliability studies (QAREL) instrument was used to assess the quality and bias of each study included in the final analysis [24]. The QAREL instrument is an 11-item system of assessment of study bias risk and quality. The scoring system used by Konieczka et al. [25] and Alfuth [14] was used to provide an overall assessment of bias risk and quality. This was conducted by two reviewers independently for the 64 articles included in the final analysis. Results were collected and refereed by a third reviewer.

### 2.7. Data Synthesis and Analysis

The utilization of two reviewers with a third reviewer for discrepancies was implemented to prevent bias for the data collection and quality and risk bias analysis. All discrepancies, including missing data by one reviewer, were mitigated with this process. Based on the prior study by Stoll et al. [26], multiple reviewers increase quality, accuracy and number of studies discovered in an SROL.

We did not perform a meta-analysis of the data for this project. However, we did follow recommendations in the synthesis without meta-analysis (SWiM) guidelines [27] for subgroup analysis to improve the certainty of outcome findings.

## 3. Results

### 3.1. Search Terms and Selection Process

A total of 2387 potential articles were discovered. Utilization of a deduplication tool in EndNote version 21 [28] resulted in the removal of 337 articles and, thus, 490 additional articles were removed after filtering titles and abstracts for the terms identified in Figure 1 of our search strategy.

#### Screening Search Results

**Figure 2:**
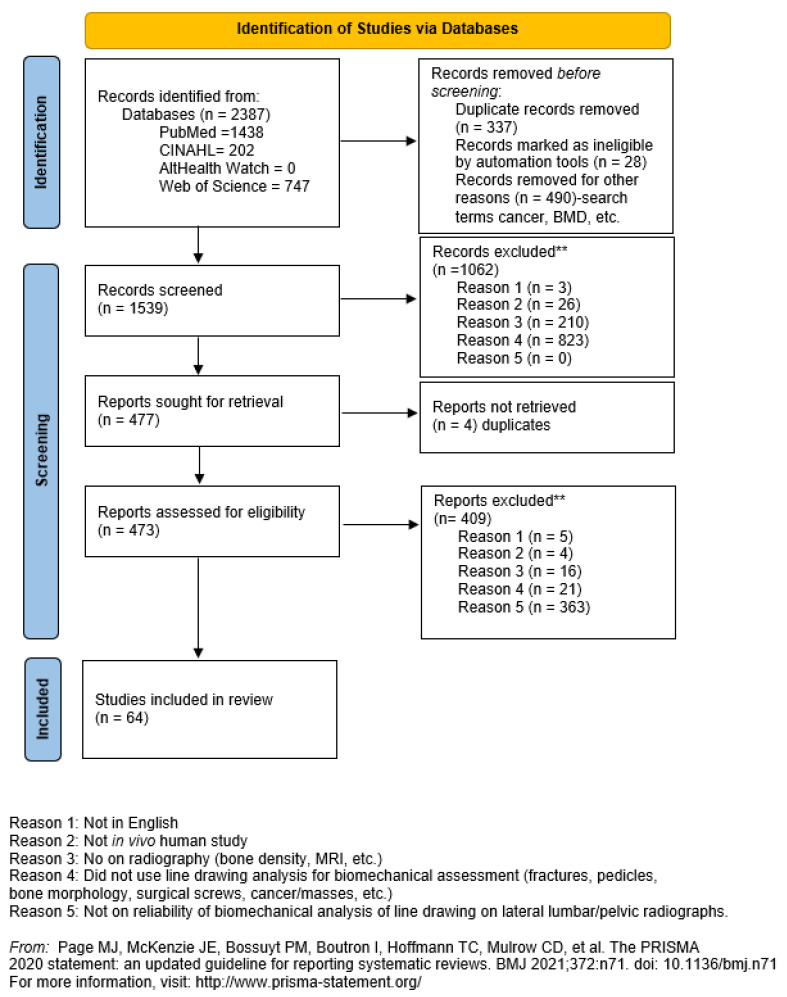
The PRISMA [22] flow diagram for the current systematic literature review.

The remaining 1539 article titles and abstracts were screened by two independent reviewers according to the inclusion and exclusion criteria and a third for discrepencies between the first two. Database records identified were PubMed (n = 1438), CINAHL (n = 202), AltHealth Watch (n = 0), and Web Of Science (n = 747). Records removed prior to screening were due to duplication (n = 337), ineligible due to automation tool usage (n = 28), and records removed for other reasons such as pathologies, anomalies, bone mineral density, etc. (n = 490). The total records removed prior to screening was 855. In total, 1062 studies were excluded by the two independent reviewers prior to retrieval request leaving 477 studies remaining. Exclusions are shown in Figure 2 of the PRISMA diagram.

The 477 remaining reports were sought for retrieval and further screening. Of these retrieved, 4 were found to be duplicates and thus excluded. The remaining 473 reports were screened by the two independent reviewers. A total of 409 studies were excluded by the two independent reviewers leaving 64 studies remaining which are included in this SROL [29,30,31,32,33,34,35,36,37,38,39,40,41,42,43,44,45,46,47,48,49,50,51,52,53,54,55,56,57,58,59,60,61,62,63,64,65,66,67,68,69,70,71,72,73,74,75,76,77,78,79,80,81,82,83,84,85,86,87,88,89,90,91,92]. Exclusions were as follows: criterion 1 (n = 5), criterion 2 (n = 4), criterion 3 (n = 16), criterion 4 (n = 21), criterion 5 (n = 363). Figure 2 shows the PRISMA [22] flow diagram for these results.

### 3.2. Study Characteristics

Table S1 contains the study characteristics and findings of the 64 included studies. Details were recorded on the study group, number of investigators, procedures for repeat analysis, methods of analysis, and stastistical results for the 64 studies.

### 3.3. Bias and Quality Analysis Using the QAREL Instrument

**Figure 3:**
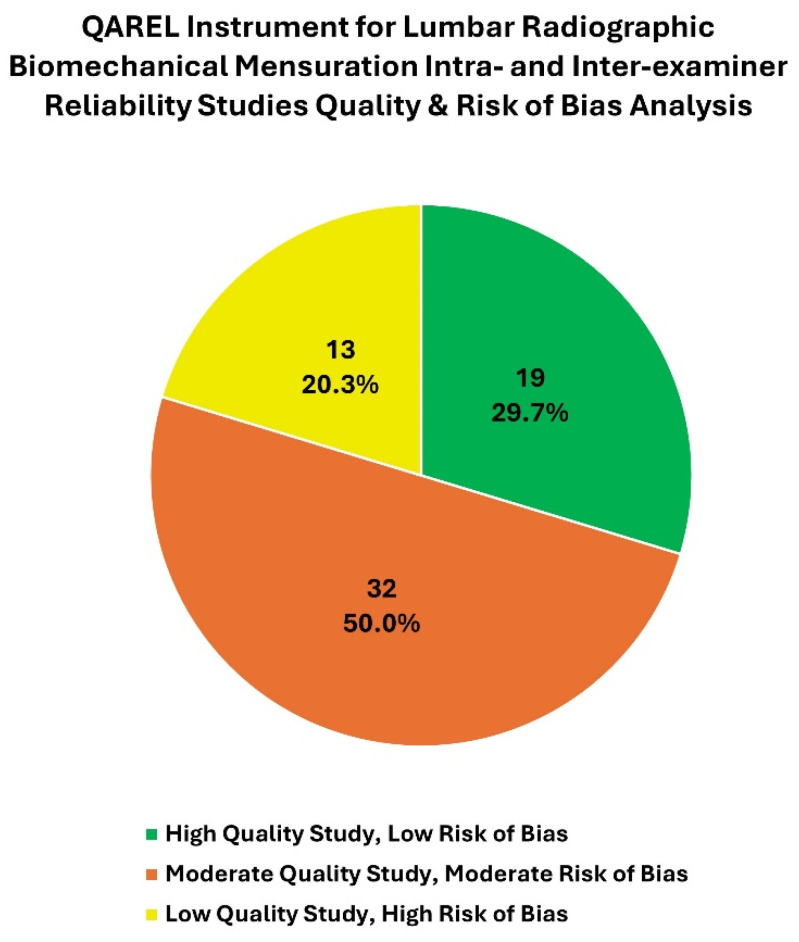
This pie chart represents the number and percentage of high-, moderate-, and low-quality (indicating low, moderate, and high risk of bias, respectively) intra- and inter-examiner reliability studies for lumbar radiographic biomechanical mensuration methods using the QAREL instrument (Table S1).

To assess the bias risk and quality of the studies, the 11-item QAREL instrument was used [24,93]. Of the 64 included articles, using the scoring system reported by Konieczka [25] and Alfuth [14], 19 studies had a low risk of bias (high quality), 32 had a moderate risk of bias (moderate quality), and 13 had a high risk of bias (low quality). See Table 1 for these data. In Figure 3, we present a visual representation of the ratings for these studies detailed in Table 1.

### 3.4. Subgroup Analysis of Similar Measurement Methods

In order to compare studies using a similar line drawing methodology for reliability assessment, we present a subgroup analysis of these studies herein. We have summarized data, including study bias analysis and statistical findings, according to studies reporting intra-examiner (Table S2) and inter-examiner (Table S3) assessment for each method of measurement reported.

**Figure 4:**
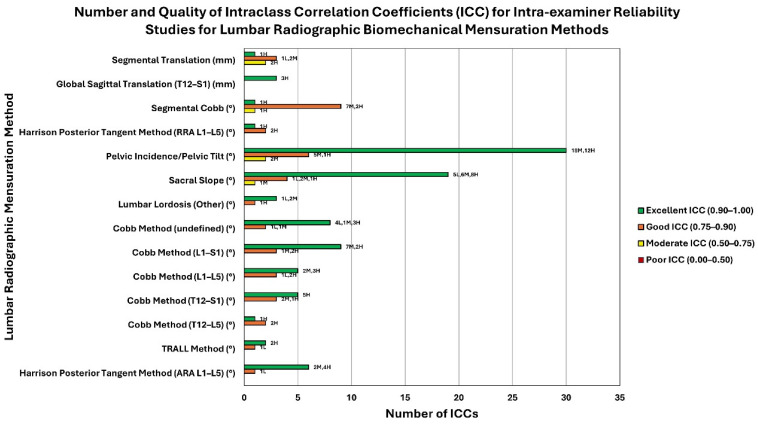
This cluster bar chart represents the quality of reliability, quality of study, and number of studies found for intra-examiner reliability of lumbar radiographic biomechanical mensuration methods (Table S2). Each mensuration method (y-axis) may have up to four bars showing the number of ICCs (x-axis) for each lumbar radiographic mensuration method (y-axis) representing the quality of ICC (excellent, good, moderate, or poor; see legend for color). The data labels at the outside end of the bars represent the number of low- (L), moderate- (M), and high-quality (H) studies (as determined using the QAREL instrument) that comprise the total number of studies for a given ICC quality (excellent, good, moderate, or poor). For example, The Harrison Posterior Tangent Method (ARA L1-L5) shows 6 excellent intra-examiner ICCs (comprised of 2 moderate-quality studies and 4 high-quality studies) and 1 good intra-examiner ICC (comprised of 1 low-quality study). The unit of measurement (° or mm) for each lumbar radiographic biomechanical mensuration method is indicated in parentheses next to the mensuration method.

**Figure 5:**
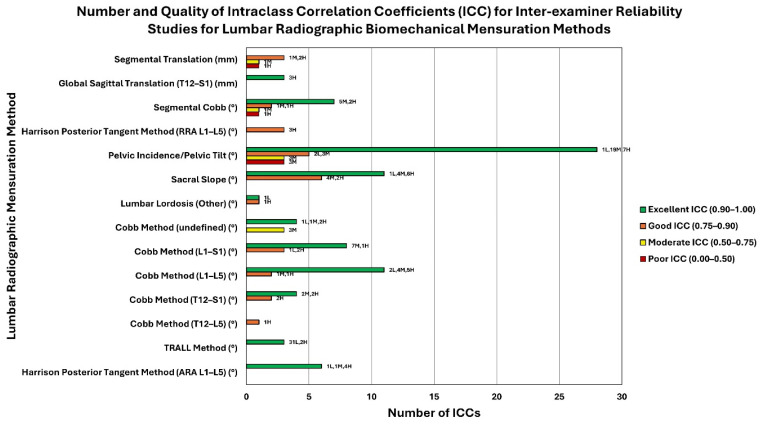
This cluster bar chart represents the quality of reliability, quality of study, and number of studies found for inter-examiner reliability of lumbar radiographic biomechanical mensuration methods (Table S3). Each mensuration method (y-axis) may have up to four bars showing the number of ICCs (x-axis) for each lumbar radiographic mensuration method (y-axis) representing the quality of ICC (excellent, good, moderate, or poor; see legend for color) The data labels at the outside end of the bars represent the number of low- (L), moderate- (M), and high-quality (H) studies (as determined using the QAREL instrument) that comprise the total number of studies for a given ICC quality (excellent, good, moderate, or poor). For example, the Harrison Posterior Tangent Method (ARA L1-L5) shows 6 excellent inter-examiner ICCs (comprised of 1 low-quality study, 2 moderate-quality studies, and 4 high-quality studies). The unit of measurement (° or mm) for each lumbar radiographic biomechanical mensuration method is indicated in parentheses next to the mensuration method.

Lumbar lordosis was the most common variable analyzed (43/64 studies). The most common method of lumbar lordosis analysis studied for reliability was the Cobb method conducted at various levels: T12–L5 (n = 1), T12–S1 (n = 7), L1–L5 (n = 11), and L1–S1 (n = 17). Nine additional studies did not report which levels were measured. Table 1 shows that intra-examiner reliability (reported in Table S2) was good to excellent for all studies that rated moderate to low risk of bias and that reported the levels studied. Inter-examiner reliability analysis ranged from good to excellent for all studies reporting the levels analyzed as shown in Table S3. The number and quality of the studies reporting intra-class correlation coefficients (ICCs) for intra-examiner reliability are shown in Figure 4. The number and quality of the studies reporting intra-class correlation coefficients (ICCs) for inter-examiner reliability are shown in Figure 5.

Intra-examiner reliability of the Harrison Posterior Tangent Method (HPTM) was investigated in 7 studies [46,49,57,71,72,82,83]. Five of the seven studies were of low or moderate bias risk (indicating high to moderate quality) [57,71,72,82,83]. These five studies included an analysis of intra-examiner reliability reporting ICCs in the excellent range (0.96–0.991), Table S2. These studies also investigated inter-examiner reliability of the HPTM and reported similar findings of excellent reliability (ICC = 0.91–0.985), Table S3.

There were 19 studies that investigated the reliability of measuring sacral slope, or sacral base angle. Intra-examiner reliability was evaluated in 15/19 of these studies and 14/15 were of low to moderate risk of bias with high to moderate quality. These investigations reported ICCs between 0.83 and 0.99, indicating good to excellent intra-examiner reliability. Table S2 reports the studies detailing intra-examiner reliability of the various methods. There were 18 studies that included an analysis of inter-examiner reliability of sacral slope. In these 18 studies, 16 were of moderate to low risk of bias indicating high to moderate quality. These studies reported ICCs ranging from 0.82 to 0.99. Table S3 reports the studies detailing inter-examiner reliability of the various methods.

**Figure 6:**
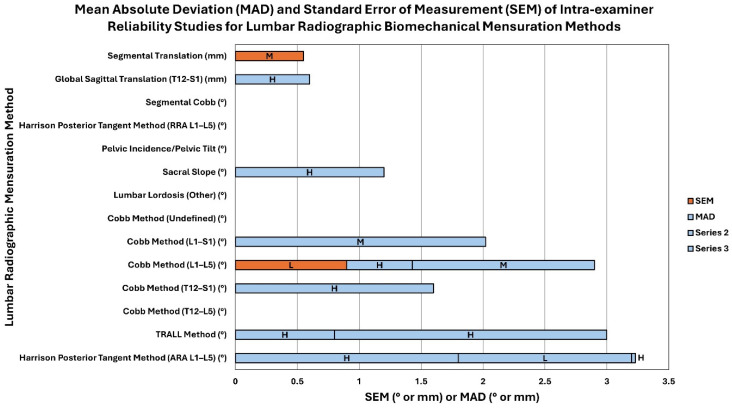
This is a stacked bar chart representing the mean absolute deviation (MAD) and standard error of measurement (SEM) found in intra-examiner reliability studies for lumbar radiographic biomechanical mensuration methods (Table S2). The stacked bars show the number values of the MAD and SEM (x-axis; see legend for color) for each lumbar radiographic mensuration method (y-axis) and the letter in each bar represents the quality of the study as determined by the QAREL instrument (L = low quality, M = moderate quality, and H = high uality). The unit of measurement (° or mm) for each lumbar radiographic biomechanical mensuration method is indicated in parentheses next to the mensuration method.

**Figure 7:**
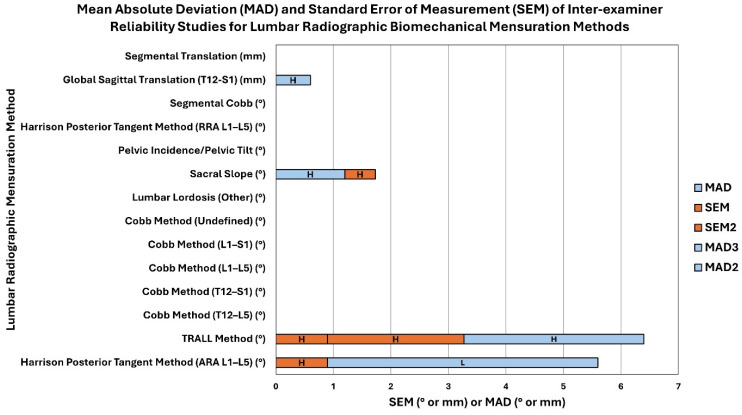
This is a stacked bar chart representing the mean absolute deviation (MAD) and standard error of measurement (SEM) found in inter-examiner reliability studies for lumbar radiographic biomechanical mensuration methods (Table S3). The stacked bars show the number values of the MAD and SEM (x-axis; see legend for color) for each lumbar radiographic mensuration method (y-axis) and the letter in each bar represents the quality of the study as determined by the QAREL instrument (L = low quality, M = moderate quality, and H = high quality). The unit of measurement (° or mm) for each lumbar radiographic biomechanical mensuration method is indicated in parentheses next to the mensuration method.

There were 21 studies that included reliability analysis of measuring pelvic morphology, including pelvic incidence, pelvic tilt, etc. Out of these 21 studies, 16 of these studies were found to have moderate to low risk of bias indicating high to moderate quality. Intra-examiner reliability of pelvic incidence ranged from 0.69 to 0.99, while inter-examiner reliability ranged from 0.69 to 0.999. Pelvic tilt measurement was found to have ICCs for intra-examiner reliability ranging from 0.41 to 0.98. Tables S2 and S3 report these data. Figure 6 and Figure 7 depict the mean absolute deviation (MAD) and standard error of measurement (SEM) found in intra-examiner and inter-examiner reliability studies, respectively.

## 4. Discussion

We hypothesized that a properly designed and executed SROL would find the majority of studies on the reliability of biomechanical analysis of sagittal lumbo-pelvic parameters on radiographs would show good to excellent reliability. Our results have confirmed this hypothesis and thus, the null hypothesis must be rejected. The result of our SROL is in stark contrast to past narrative reviews and rapid reviews with narrower inclusion criteria [15,16,17,18,19]. The most studied parameter of reliability of sagittal lumbo-pelvic biomechanical assessment of radiographs was the magnitude of lumbar lordosis, followed by sacral slope (sacral base angle), pelvic morphology (pelvic incidence, pelvic tilt, etc.), segmental rotation and translation measurements, and regional sagittal balance/sagittal translation of T12 relative to S1. Furthermore, the most investigated methodology for measurement of sagittal lumbo-pelvic lordosis is the Cobb method at various levels followed by the HPTM, then the TRALL method, and the Centroid and other methodologies. Considering the evidence for each of these measurement methods, all of them have at least one high-quality intra-examiner reliability investigation demonstrating good to excellent reliability (range of 1–6 studies pending the methodology and measurement levels) and at least one high-quality inter-examiner reliability investigation demonstrating good to excellent reliability (range of 1–6 studies pending the methodology and measurement levels). Review Tables S2 and S3 for details.

### 4.1. Heterogeneity of Findings

A primary finding of our SROL is that the majority of the radiographic measurement techniques studied showed consistent good to excellent reliability. However, there were some measures that showed significant heterogeneity in results. For example, two of the reports investigating the reliability of pelvic incidence reported a broad range of ICCs [48,55]. Upon inspection of the papers, these were investigations comparing manual vs. computer-aided methods of measuring pelvic incidence and pelvic tilt [48,55]. While the manual methods showed lower reliability, the investigators found excellent reliability using the computer-aided methods. Other sources of heterogeneity in the results can be explained by the type (quality) of the image used (digital vs. plain film), source type (X-ray, biplanar stereoradiography, EOS), different statistical reporting (ICC, Pearson, Kappa, Bland–Altman, etc.) and studies including varying spinal pathologies that make identification of anatomical landmarks more difficult (spondylosis, osteoarthritis, etc.). These sources of heterogeneity prevented us from performing a more thorough meta-analysis of the data.

### 4.2. Clinical Interpretation and Relevance of This Systematic Review

The current SROL demonstrating good to excellent intra- and inter-examiner reliability in the radiographic assessments for biomechanical structures in the sagittal lumbo-pelvic human spine is the first one in the literature to our knowledge on this topic. This SROL was performed according to PRISMA checklist guidelines and was undertaken primarily for clinicians who manage patients with lumbar spine conditions such as chronic low back pain (CLPB), as well as healthcare providers who utilize treatment which seeks to change sagittal lumbo-pelvic alignment (whether surgically or through rehabilitation approaches). Reliability of radiographic mensuration is important for spine biomechanical understanding, clinical interpretation, and treatment decision-making [94]. The understanding of normal and abnormal spinal biomechanical structure is important in clinical practice for primary care providers [95], chiropractors [9,11,94], physical therapists [11,96], and surgeons [2,3,4,10,97] as well as medical device manufacturers [97], spinal modeling through machine learning [4], third-party payors [98], investigational institutions [99], and governmental bodies [100].

Studies investigating the global burden of diseases (GBD) have demonstrated that spinal abnormalities that lead to pain, especially low back pain, cause dysfunction and disability and are the greatest contributor to the years lived with disability (YLDs) globally with 577 million people worldwide suffering from CLBP in 2020 [100] and is projected to increase in the under 40 years of age population by 2050 [1]. Given this significant contribution of CLBP and chronic widespread spinal pain (CWSP) to individual and GBD, understanding the causes, consequences, and symptoms is important across many healthcare disciplines [1,100,101]. Clinician certainty of reliability of diagnostic methods should obviously improve physical interventions and their outcomes and this has recently been documented for conservative interventions aimed at improving the sagittal lumbo-pelvic lordosis using radiographic measurements as a primary diagnostic tool and outcome of care [11,102,103]. Beyond conservative therapeutic interventions, it is of the utmost diagnostic necessity to have valid, reliable, and repeatable methods to assess spinal biomechanics, lumbo-pelvic in particular, for invasive interventions such as spinal surgery for deformity [2,3,4,11,104,105]. There is promising development of machine learning and artificial intelligence software programs that have extremely high reliability when measuring biomechanical abnormalities. Future studies using computer vision and machine learning will likely increase the clinical certainty for spine disorders diagnosis and treatment [106,107].

Obviously, spine deformities and other musculoskeletal disorders that lead to chronic pain and disability have numerous causes and are not just influenced by spinal alignment variables. Known contributors to chronic pain and disability include anatomical [108], morphological [109], genetic [110], traumatic [111], postural [112], severe stress [113], psychosocial [114], and biomechanical [115], including altered sagittal lumbar alignment [9,10,11,12,13]). Modern clinicians must take into consideration each of the potential causes of a patient’s pain and suffering, and this diagnostic clinical certainty must have proper objective and reliable outcome measures. It is our contention that proper radiographic measurement methods for the assessment of spine displacement must be included in the clinical armamentarium of today’s spine clinicians.

### 4.3. Flaws With Previous Non-Systematic Reviews

Interestingly, the results of our SROL are at extreme odds with several non-systematic reviews of the literature on radiography usage in conservative spine care including a critical commentary on the use of radiography [15], a proposed diagnostic imaging guide [16], a rapid literature review on radiography [18], and two narrative reviews on radiography [17,19]. Three main themes arise in these previous reviews [15,16,17,18,19] that can be categorized into the following items: (1) radiation carries with it a high risk of injury (cancer risk) based on the linear no-threshold (LNT) model of radiation exposure; (2) X-ray line drawing for spine displacement analysis and interventions lack clear reliability; and (3) spine interventions designed to reduce, improve, or correct radiographic-measured displacements lack clinical utility and validity. In regard to the first critical theme, recent reports demonstrate a clear presentation that radiography is safe and the doses which are used in plain film radiography are miniscule compared to other common sources of radiation [116,117,118]. Exposure to ionizing radiation does cause cellular (DNA fragment) damage via free radical production; however, the amount of damage is miniscule (1/1-millionth) in the scheme of normal metabolic daily processes [117]. Second, reliance on the LNT model is not scientific [119,120]; it is anti-evolutionary [121] and invalid [122]. Recently, the LNT hypothesis has been found to be shrouded in controversy as the basis relies on the fatally flawed Muller experiments [123,124]. Finally, a recent systematic review [125] of articles published from 1975 to 2017 examined cancer risk from external low-dose X-ray and gamma radiation (<200 mSv). In a detailed review of the initial 62 articles, only 25 studies met higher-quality criteria and of these, 21 out of 25 studies did not support cancer induction by low-dose exposure to radiation (*p* = 0.0003) [118].

Regarding theme two above, in general, the conflicting findings between our SROL and these other reviews point to the inherent biases (opinions of authors and selection bias) of the critical commentary [15] and narrative reviews [17,19] and the limitations with the methodology of article inclusion (selection bias) in the rapid review [18]. Our analysis of the 64 included studies herein on sagittal lumbar line drawing reliability clearly establishes these measurements as highly reliable in clinical practice for the assessment of spine displacements and deformities. Importantly, the overt flaws with two of these reviews [17,18] have been previously detailed [21,125]; however, it is surprising how far reaching the consequences (the misuse) of a flawed review can be [20]; the restriction of X-ray rights for Chiropractors in British Columbia occurred as a result.

It might be argued that the criticism expressed in the third theme above is the most pressing issue concerning the clinical utility of patient-relevant outcomes for X-ray-based spine care. The reliability of radiographic measurement methods is a cornerstone of proper spine evaluation governing the selection of proper intervention strategies in both conservative and surgical-based spine outcomes [2,3,5,10,11,102,103,106,117,126]. Given the seriousness of the GBD for low back pain-related disorders [1,100], this issue of flawed and/or biased reviews becomes critically important in light of the need for better-quality diagnosis and interventions using conservative care for lumbar spine disorders. Importantly, recent conservative care studies have identified that patients receiving care designed to improve their altered sagittal plane lumbo-pelvic spine alignment (as identified and quantified using X-ray measurement methods) offer considerable benefit in the form of improved pain intensity, disability, function, and neurophysiology [11,102,103,126]. Similarly, recent surgical interventions have documented a clear benefit in patients with a variety of lumbar spine disorders for restoration of the sagittal lumbo-pelvic alignment towards normative alignment as measured by spine radiographic line drawing methods [2,3,5,10,106].

### 4.4. Strengths of Our SROL

Our SROL has several significant strong points based on our robust methodology. First, our review followed the recommendations of the preferred reporting items for systematic reviews and meta-analyses (PRISMA) and our design was registered with PROSPERO. Second, we performed a rather exhaustive literature search using multiple databases from the date of database inception through 4 January 2023. Thirdly, our search strategy yielded a comprehensive 64 articles that met a rigorous inclusion/exclusion criteria, and this high number of included articles is rare for the average SROL. A fourth strength of our SROL is that we followed the SWiM reporting guidelines by presenting subgroup analysis [27] where we grouped like methods of measurement for both intra- and inter-examiner reliability. Finally, the use of two separate examiners to independently evaluate and rate the included studies and a third examiner for consensus or to resolve conflict is an important feature of our design. Based on these design features, our finding that the preponderance of evidence shows good to excellent intra- and inter-examiner reliability for biomechanical assessment of sagittal lumbo-pelvic spine alignment would logically be quite robust. The results of this SROL rather conclusively demonstrate that sagittal radiographic analysis of spinal biomechanics and alignment of the human lumbo-pelvic spine is a reliable tool for aiding diagnosis and management in clinical settings.

### 4.5. Limitations

A limitation of this systematic literature review is the focus on sagittal lumbo-pelvic images only. Future studies should incorporate reviews of the remaining spine regions (cervical and thoracic) as well as full-spine lateral and A-P radiographs to determine the reliability of radiographic mensuration for spine alignment. Future systematic reviews on the validity of these measures in clinical practice need to be performed. Larger studies based on the outcomes of these systematic reviews can guide treatment options.

## 5. Conclusions

We present the first systematic literature review (SROL) on the reliability of sagittal radiographic analysis of spinal biomechanics and alignment of the human lumbo-pelvic spine to assist clinicians in understanding the use of these diagnostic tools. After a detailed exclusion, 64 articles met the inclusion criteria for this SROL. The most investigated methodology for measurement of sagittal lumbo-pelvic lordosis is the Cobb method at various levels followed by the Harrison Posterior Tangent Method, then the TRALL method, the Centroid, and other methodologies. Clinical usage of radiography for diagnosis of spine displacement is reliable in the assessment of patients suffering from spine pain and related spinal conditions.

## Figures and Tables

**Table 1:** The quality appraisal tool for studies of diagnostic reliability (QAREL)-checklist for the evaluation of quality of the included reliability studies on clinical assessments for the biomechanical analysis of the sagittal lumbar spine or pelvis on lateral radiographs (n = 64).

Study	QAREL Instrument Questions											Sum	Bias Risk	Quality	Reliability
	1	2	3	4	5	6	7	8	9	10	11				
Abdel 2012 [29]	Y	Y	Y	Y	Y	Y	N	U	U	Y	Y	8	L	H	B
Ames 2015 [30]	Y	Y	U	U	NA	Y	U	U	Y	Y	Y	6	M	M	B
Andreason 2007 [31]	Y	Y	Y	Y	NA	U	U	Y	Y	Y	Y	8	L	H	B
Bagheri 2018 [32]	Y	N	U	U	NA	U	U	U	Y	Y	Y	4	H	L	B
Bolesta 2010 [33]	Y	Y	Y	Y	NA	U	U	U	Y	Y	Y	7	M	M	B
Bredow 2015 [34]	Y	Y	Y	Y	NA	U	U	U	Y	Y	Y	7	M	M	B
Breen 2019 [35]	Y	Y	Y	Y	NA	Y	Y	U	Y	Y	Y	9	L	H	IR
Cakir 2006 [36]	Y	Y	U	U	NA	U	U	U	Y	Y	Y	5	M	M	B
Chanplakorn 2011 [37]	Y	Y	U	U	NA	U	U	U	U	Y	Y	4	H	L	IR
Chen 1999 [38]	Y	Y	Y	Y	NA	U	U	U	Y	Y	Y	7	M	M	B
Chung 2017 [39]	Y	Y	Y	Y	NA	Y	Y	Y	U	Y	Y	9	L	H	B
de Carvalho 2010 [40]	Y	Y	Y	U	NA	U	U	U	U	Y	N	4	H	L	B
Dimar 2008 [41]	Y	Y	U	U	NA	U	U	U	Y	Y	Y	5	M	M	B
du Rose 2016 [42]	Y	Y	Y	Y	NA	U	U	U	Y	Y	Y	7	M	M	B
Fritz 2005 [43]	U	Y	Y	Y	NA	Y	Y	Y	Y	Y	Y	9	L	H	IA
Gilliam 1994 [44]	Y	Y	Y	U	NA	U	U	U	U	Y	Y	5	M	M	B
Gladnick 2017 [45]	Y	Y	Y	NA	Y	Y	U	NA	Y	Y	Y	8	L	H	IR
Harrison 2001 [46]	Y	Y	Y	NA	Y	U	U	Y	Y	Y	Y	8	L	H	B
Hicks 2006 [47]	Y	Y	Y	NA	NA	U	U	N	NA	Y	Y	5	M	M	IR
Hohenhaus 2022 [48]	Y	Y	Y	NA	NA	U	U	NA	Y	Y	Y	6	M	M	IR
Hong 2010 [49]	Y	Y	Y	Y	NA	U	U	Y	Y	Y	Y	8	L	H	B
Jackson 2000 [50]	Y	Y	Y	Y	NA	U	U	U	Y	Y	Y	7	M	M	B
Jackson 1998 [51]	Y	Y	Y	Y	NA	U	U	U	U	Y	Y	6	M	M	B
Karabag 2022 [52]	Y	Y	U	U	NA	U	U	U	U	Y	Y	4	H	L	B
Kepler 2015 [53]	Y	Y	Y	Y	NA	Y	Y	Y	Y	Y	Y	10	L	H	B
Khalsa 2018 [54]	Y	Y	Y	NA	NA	U	U	U	Y	N	Y	5	M	M	B
Kunkle 2017 [55]	Y	Y	Y	Y	NA	N	U	U	U	Y	Y	6	M	M	B
Lafage 2015 [56]	Y	Y	Y	Y	Y	Y	U	Y	Y	Y	Y	10	L	H	B
Lee 2019 [57]	Y	Y	U	U	NA	U	U	U	Y	Y	Y	5	M	M	B
Lee 2013 [58]	Y	Y	U	U	NA	U	U	U	Y	Y	Y	5	M	M	B
Marchetti 2007 [59]	Y	Y	Y	Y	NA	Y	U	Y	Y	Y	Y	9	L	H	B
McCarty 2009 [60]	Y	Y	Y	Y	NA	U	U	U	Y	Y	Y	7	M	M	B
Mellor 2014 [61]	Y	Y	U	U	NA	U	U	U	Y	Y	Y	5	M	M	B
Newton 2016 [62]	Y	Y	U	U	NA	U	U	U	Y	Y	Y	5	M	M	B
Okpala 2018 [63]	Y	U	U	U	NA	U	U	U	U	Y	N	2	H	L	IR
Orosz 2022 [64]	Y	Y	Y	NA	NA	U	U	U	NA	Y	Y	5	M	M	IR
Pearson 2011 [65]	Y	Y	Y	Y	NA	Y	Y	Y	U	Y	Y	9	L	H	B
Pinel-Giroux 2006 [66]	Y	Y	U	U	NA	U	U	U	Y	Y	Y	5	M	M	B
Plaugher 1990 [67]	Y	Y	Y	Y	NA	Y	N	U	U	Y	Y	7	M	M	B
Polly 1996 [68]	Y	Y	Y	Y	NA	Y	Y	Y	Y	Y	Y	10	L	H	B
Rastegar 2018 [69]	Y	Y	U	Y	U	U	U	Y	Y	Y	Y	7	M	M	B
Rehm 2017 [70]	Y	U	U	U	NA	U	U	U	U	Y	Y	3	H	L	IR
Ruhinda 2014 [71]	Y	Y	U	U	NA	U	U	U	U	N	N	2	H	L	B
Russell 2020 [72]	Y	Y	Y	U	NA	U	U	U	U	Y	Y	5	M	M	B
Segundo 2016 [73]	Y	Y	Y	U	NA	U	U	U	U	Y	Y	5	M	M	IR
Severijns 2020 [74]	Y	Y	Y	U	NA	U	U	U	Y	Y	Y	6	M	M	B
Suzuki 2010 [75]	Y	Y	U	U	U	U	U	U	Y	Y	Y	5	M	M	B
Suzuki 2020 [76]	Y	Y	U	U	U	U	U	U	Y	Y	Y	5	M	M	B
Taghipour-Darzi 2009 [77]	Y	Y	NA	U	U	U	U	U	Y	Y	Y	5	M	M	IA
Takahashi 2021 [78]	Y	Y	Y	Y	U	U	U	Y	Y	Y	Y	8	L	H	B
Tallroth 1994 [79]	Y	Y	Y	Y	U	U	U	Y	Y	Y	Y	8	L	H	B
Teyhen 2005 [80]	Y	Y	U	U	U	U	U	Y	Y	Y	Y	6	M	M	IA
Timon 2005 [81]	Y	Y	U	Y	U	U	U	Y	Y	Y	Y	7	M	M	B
Troyanovich 1995 [82]	Y	Y	Y	Y	U	U	U	Y	Y	Y	Y	8	L	H	B
Troyanovich 1998 [83]	Y	Y	Y	Y	U	U	U	Y	Y	Y	Y	8	L	H	B
Wang 2010 [84]	Y	Y	U	U	U	U	U	U	Y	Y	Y	5	M	M	B
Wanke-Jellinek 2019 [85]	Y	Y	U	NA	U	U	U	U	NA	Y	Y	4	H	L	IR
Wong 2019 [86]	Y	Y	U	U	U	U	U	U	U	Y	Y	4	H	L	IA
Wu 2014 [87]	Y	Y	Y	Y	U	U	U	Y	Y	Y	Y	8	L	H	IA
Wu 2021 [88]	Y	Y	U	U	U	U	U	U	U	Y	Y	4	H	L	B
Zhang 2022 [89]	Y	U	NA	U	NA	U	U	U	U	Y	Y	3	H	L	IA
Zhou 2021 [90]	Y	Y	NA	U	U	U	U	U	U	Y	Y	4	H	L	IA
Zhou 2022 [91]	Y	Y	Y	U	U	U	U	N	Y	Y	Y	6	M	M	B
Zhu 2015 [92]	Y	Y	Y	U	U	U	U	U	Y	Y	Y	6	M	M	B

Note: N = No; Y = Yes; U = Unclear; NA = Not applicable; H = High; M = Moderate; L = Low; IA = Intra-examiner reliability; IR = Inter-examiner reliability; B = Both Intra- and Inter-examiner reliability.

## Data Availability

These data were derived from the resources available in the public domain. The authors declare that this literature review is not based on original data.

## Supplementary Materials

The following supporting information can be downloaded at: https://www.mdpi.com/article/10.3390/jcm13164650/s1.

## References

[1] MSK in Adolescents Collaborators (2024). Global pattern, trend, and cross-country inequality of early musculoskeletal disorders from 1990 to 2019, with projection from 2020 to 2050. Med.

[2] Rothenfluh D.A., Mueller D.A., Rothenfluh E., Min K. (2015). Pelvic incidence-lumbar lordosis mismatch predisposes to adjacent segment disease after lumbar spinal fusion. Eur. Spine J..

[3] Hey H.W.D., Tan K.A., Kantharajanna S.B., Teo A.Q.A., Chan C.X., Liu K.G., Wong H.K. (2018). Using spinopelvic parameters to estimate residual lumbar lordosis assuming previous lumbosacral fusion-a study of normative values. Spine J..

[4] Harada G.K., Siyaji Z.K., Younis S., Louie P.K., Samartzis D., An H.S. (2019). Imaging in spine surgery: Current concepts and future directions. Spine Surg. Relat. Res..

[5] Rubery P.T., Lander S.T., Mesfin A., Sanders J.O., Thirukumaran C.P. (2022). Mismatch between pelvic incidence and lumbar lordosis is the key sagittal plane determinant of patient outcome at minimum 40 years after instrumented fusion for adolescent idiopathic scoliosis. Spine.

[6] Guo J., Liu Z., Lv F., Zhu Z., Qian B., Zhang X., Lin X., Sun X., Qiu Y. (2012). Pelvic tilt and trunk inclination: New predictive factors in curve progression during the Milwaukee bracing for adolescent idiopathic scoliosis. Eur. Spine J..

[7] Catanzano A.A., Esposito V.R., Dial B.L., Wu C.J., Hinton Z.W., Risoli T.J., Green C.L., Fitch R.D., Lark R.K. (2020). Staying ahead of the curve: The use of spinopelvic parameters to predict curve progression and bracing success in adolescent idiopathic scoliosis. Spine Deform..

[8] Landauer F., Trieb K. (2022). Scoliosis: Brace treatment—From the past 50 years to the future. Medicine.

[9] Harrison D.E., Haas J.W., Moustafa I.M., Betz J.W., Oakley P.A. (2024). Can the mismatch of measured pelvic morphology vs. lumbar lordosis predict chronic low back pain patients?. J. Clin. Med..

[10] Diebo B.G., Varghese J.J., Lafage R., Schwab F.J., Lafage V. (2015). Sagittal alignment of the spine: What do you need to know?. Clin. Neurol. Neurosurg..

[11] Oakley P.A., Ehsani N.N., Moustafa I.M., Harrison D.E. (2020). Restoring lumbar lordosis: A systematic review of controlled trials utilizing Chiropractic Bio Physics^®^ (CBP^®^) non-surgical approach to increasing lumbar lordosis in the treatment of low back disorders. J. Phys. Ther. Sci..

[12] Chun S.W., Lim C.Y., Kim K., Hwang J., Chung S.G. (2017). The relationships between low back pain and lumbar lordosis: A systematic review and meta-analysis. Spine J..

[13] Sadler S.G., Spink M.J., Ho A., De Jonge X.J., Chuter V.H. (2017). Restriction in lateral bending range of motion, lumbar lordosis, and hamstring flexibility predicts the development of low back pain: A systematic review of prospective cohort studies. BMC Musculoskelet. Disord..

[14] Alfuth M., Fichter P., Knicker A. (2021). Leg length discrepancy: A systematic review on the validity and reliability of clinical as-sessments and imaging diagnostics used in clinical practice. PLoS ONE.

[15] Bussieres A.E., Ammendolia C., Peterson C., Taylor J.A. (2006). Ionizing radiation exposure—more good than harm? The preponderance of evidence does not support abandoning current standards and regulations. J. Can. Chiropr. Assoc..

[16] Bussieres A.E., Taylor J.A., Peterson C. (2008). Diagnostic imaging practice guidelines for musculoskeletal complaints in adults-an evidence-based approach-part 3: Spinal disorders. J. Manip. Physiol. Ther..

[17] Jenkins H.J., Downie A.S., Moore C.S., French S.D. (2018). Current evidence for spinal X-ray use in the chiropractic profession: A narrative review. Chiropr. Man. Therap.

[18] Corso M., Cancelliere C., Mior S., Kumar V., Smith A., Cote P. (2020). The clinical utility of routine spinal radiographs by chiropractors: A rapid review of the literature. Chiropr. Man. Therap.

[19] Young K.J., Bakkum B.W., Siordia L. (2016). The Hangover: The Early and Lasting Effects of the Controversial Incorporation of X-Ray Technology into Chiropractic. Health Hist..

[20] College Board of Chiropractors of British Columbia (2021). Amendments to the PCH: Routine and Repeat Imaging. Professional Conduct Handbook.

[21] Oakley P.A., Betz J.W., Harrison D.E., Siskin L.A., Hirsh D.W. (2021). International chiropractors association rapid response research review. Radiophobia overreaction: College of Chiropractors of British Columbia revoke full X-ray rights based on flawed study and radiation fear-mongering. Dose Response.

[22] Page M.J., McKenzie J.E., Bossuyt P.M., Boutron I., Hoffmann T.C., Mulrow C.D., Shamseer L., Tetzlaff J.M., Akl E.A., Brennan S.E. (2021). The PRISMA 2020 statement: An updated guideline for reporting systematic reviews. Syst. Rev..

[23] McGowan J., Sampson M., Salzwedel D.M., Cogo E., Foerster V., Lefebvre C. (2016). PRESS peer review of electronic search strate-gies: 2015 guideline statement. J. Clin. Epidemiol..

[24] Lucas N., Macaskill P., Irwig L., Moran R., Rickards L., Turner R., Bogduk N. (2013). The reliability of a quality appraisal tool for studies of diagnostic reliability (QAREL). BMC Med. Res. Methodol..

[25] Konieczka C., Gibson C., Russett L., Dlot L., MacDermid J., Watson L., Sadi J. (2017). What is the reliability of clinical measurement tests for humeral head position? A systematic review. J. Hand Ther..

[26] Stoll C.R.T., Izadi S., Fowler S., Green P., Suls J., Colditz G.A. (2019). The value of a second reviewer for study selection in systematic reviews. Res. Synth. Methods.

[27] Campbell M., McKenzie J.E., Sowden A., Katikireddi S.V., Brennan S.E., Ellis S., Hartmann-Boyce J., Ryan R., Shepperd S., Thomas J. (2020). Synthesis without meta-analysis (SWiM) in systematic reviews: Reporting guideline. BMJ.

[28] Barnett I., Malik N., Kuijjer M.L., Mucha P.J., Onnela J.P. (2019). Endnote: Feature-based classification of networks. Netw. Sci..

[29] Abdel M.P., Bodemer W.S., Anderson P.A. (2012). Supine thoracolumbar sagittal spine alignment: Comparing computerized tomography and plain radiographs. Spine.

[30] Ames C.P., Smith J.S., Eastlack R., Blaskiewicz D.J., Shaffrey C.I., Schwab F., Bess S., Kim H.J., Mundis G.M., Klineberg E. (2015). Reliability assessment of a novel cervical spine deformity classification system. J. Neurosurg. Spine.

[31] Andreasen M.L., Langhoff L., Jensen T.S., Albert H.B. (2007). Reproduction of the lumbar lordosis: A comparison of standing radiographs versus supine magnetic resonance imaging obtained with straightened lower extremities. J. Manip. Physiol. Ther..

[32] Bagheri A., Liu X.C., Tassone C., Thometz J., Tarima S. (2018). Reliability of three-dimensional spinal modeling of patients with idiopathic scoliosis using EOS system. Spine Deform..

[33] Bolesta M.J., Winslow L., Gill K. (2010). A comparison of film and computer workstation measurements of degenerative spondylolisthesis: Intraobserver and interobserver reliability. Spine.

[34] Bredow J., Oppermann J., Scheyerer M.J., Gundlfinger K., Neiss W.F., Budde S., Floerkemeier T., Eysel P., Beyer F. (2015). Lumbar lordosis and sacral slope in lumbar spinal stenosis: Standard values and measurement accuracy. Arch. Orthop. Trauma. Surg..

[35] Breen A., Hemming R., Mellor F., Breen A. (2019). Intrasubject repeatability of in vivo intervertebral motion parameters using quantitative fluoroscopy. Eur. Spine J..

[36] Cakir B., Richter M., Käfer W., Wieser M., Puhl W., Schmidt R. (2006). Evaluation of lumbar spine motion with dynamic X-ray—A reliability analysis. Spine.

[37] Chanplakorn P., Wongsak S., Woratanarat P., Wajanavisit W., Laohacharoensombat W. (2011). Lumbopelvic alignment on standing lateral radiograph of adult volunteers and the classification in the sagittal alignment of lumbar spine. Eur. Spine J..

[38] Chen Y.L. (1999). Vertebral centroid measurement of lumbar lordosis compared with the Cobb technique. Spine.

[39] Chung N.S., Jeon C.H., Lee H.D., Won S.H. (2017). Measurement of spinopelvic parameters on standing lateral lumbar radiographs: Validity and reliability. Clin. Spine Surg..

[40] De Carvalho D.E., Soave D., Ross K., Callaghan J.P. (2010). Lumbar spine and pelvic posture between standing and sitting: A radiologic investigation including reliability and repeatability of the lumbar lordosis measure. J. Manip. Physiol. Ther..

[41] Dimar J.R., Carreon L.Y., Labelle H., Djurasovic M., Weidenbaum M., Brown C., Roussouly P. (2008). Intra- and inter-observer reliability of determining radiographic sagittal parameters of the spine and pelvis using a manual and a computer-assisted methods. Eur. Spine J..

[42] du Rose A., Breen A. (2016). Relationships between lumbar inter-vertebral motion and lordosis in healthy adult males: A cross sectional cohort study. BMC Musculoskelet. Disord..

[43] Fritz J.M., Piva S.R., Childs J.D. (2005). Accuracy of the clinical examination to predict radiographic instability of the lumbar spine. Eur. Spine J..

[44] Gilliam J., Brunt D., MacMillan M., Kinard R.E., Montgomery W.J. (1994). Relationship of the pelvic angle to the sacral angle: Measurement of clinical reliability and validity. J. Orthop. Sports Phys. Ther..

[45] Gladnick B.P., Schreiber J.J., Ishmael C.R., Bjerke-Kroll B.T., Cunningham M.E. (2017). Assessment of vertebral curves using the manual post-it technique. Clin. Spine Surg..

[46] Harrison D.E., Harrison D.D., Cailliet R., Janik T.J., Holland B. (2001). Radiographic analysis of lumbar lordosis: Centroid, Cobb, TRALL, and Harrison posterior tangent methods. Spine.

[47] Hicks G.E., George S.Z., Nevitt M.A., Cauley J.A., Vogt M.T. (2006). Measurement of lumbar lordosis: Inter-rater reliability, minimum detectable change and longitudinal variation. J. Spinal Disord. Tech..

[48] Hohenhaus M., Volz F., Merz Y., Watzlawick R., Scholz C., Hubbe U., Klingler J.H. (2022). The challenge of measuring spinopelvic parameters: Inter-rater reliability before and after minimally invasive lumbar spondylodesis. BMC Musculoskelet. Disord..

[49] Hong J.Y., Suh S.W., Modi H.N., Hur C.Y., Song H.R., Park J.H. (2010). Reliability analysis for radiographic measures of lumbar lordosis in adult scoliosis: A case-control study comparing 6 methods. Eur. Spine J..

[50] Jackson R.P., Kanemura T., Kawakami N., Hales C. (2000). Lumbopelvic lordosis and pelvic balance on repeated standing lateral radiographs of adult volunteers and untreated patients with constant low back pain. Spine.

[51] Jackson R.P., Peterson M.D., McManus A.C., Hales C. (1998). Compensatory spinopelvic balance over the hip axis and better reliability in measuring lordosis to the pelvic radius on standing lateral radiographs of adult volunteers and patients. Spine.

[52] Karabag H., Iplikcioglu A.C., Dusak A., Karayol S.S. (2022). Pelvic incidence measurement with supine magnetic resonance imaging: A validity and reliability study. Clin. Neurol. Neurosurg..

[53] Kepler C.K., Hilibrand A.S., Sayadipour A., Koerner J.D., Rihn J.A., Radcliff K.E., Vaccaro A.R., Albert T.J., Anderson D.G. (2015). Clinical and radiographic degenerative spondylolisthesis (CARDS) classification. Spine J..

[54] Khalsa A.S., Mundis G.M., Yagi M., Fessler R.G., Bess S., Hosogane N., Park P., Than K.D., Daniels A., Iorio J. (2018). Variability in assessing spinopelvic parameters with lumbosacral transitional vertebrae: Inter- and intraobserver reliability among spine surgeons. Spine.

[55] Kunkle W.A., Madden M., Potts S., Fogelson J., Hershman S. (2017). Validity of a smartphone protractor to measure sagittal parameters in adult spinal deformity. Spine J..

[56] Lafage R., Ferrero E., Henry J.K., Challier V., Diebo B., Liabaud B., Lafage V., Schwab F. (2015). Validation of a new computer-assisted tool to measure spino-pelvic parameters. Spine J..

[57] Lee J.S., Goh T.S., Park S.H., Lee H.S., Suh K.T. (2013). Radiographic measurement reliability of lumbar lordosis in ankylosing spondylitis. Eur. Spine J..

[58] Lee J.B., Kim I.S., Lee J.J., Park J.H., Cho C.B., Yang S.H., Sung J.H., Hong J.T. (2019). Validity of a smartphone application (Sagittalmeter Pro) for the measurement of sagittal balance parameters. World Neurosurg..

[59] Marchetti B.V., Candotti C.T., Raupp E.G., Oliveira E.B.C., Furlanetto T.S., Loss J.F. (2017). Accuracy of a radiological evaluation method for thoracic and lumbar spinal curvatures using spinous processes. J. Manip. Physiol. Ther..

[60] McCarty M.E., Mehlman C.T., Tamai J., Do T.T., Crawford A.H., Klein G. (2009). Spondylolisthesis: Intraobserver and interobserver reliability with regard to the measurement of slip percentage. J. Pediatr. Orthop..

[61] Mellor F.E., Thomas P.W., Thompson P., Breen A.C. (2014). Proportional lumbar spine inter-vertebral motion patterns: A comparison of patients with chronic, non-specific low back pain and healthy controls. Eur. Spine J..

[62] Newton P.O., Khandwala Y., Bartley C.E., Reighard F.G., Bastrom T.P., Yaszay B. (2016). New EOS imaging protocol allows a substantial reduction in radiation exposure for scoliosis patients. Spine Deform..

[63] Okpala F.O. (2018). Comparison of four radiographic angular measures of lumbar lordosis. J. Neurosci. Rural. Pract..

[64] Orosz L.D., Bhatt F.R., Jazini E., Dreischarf M., Grover P., Grigorian J., Roy R., Schuler T.C., Good C.R., Haines C.M. (2022). Novel artificial intelligence algorithm: An accurate and independent measure of spinopelvic parameters. J. Neurosurg. Spine.

[65] Pearson A.M., Spratt K.F., Genuario J., McGough W., Kosman K., Lurie J., Sengupta D.K. (2011). Precision of lumbar intervertebral measurements: Does a computer-assisted technique improve reliability?. Spine.

[66] Pinel-Giroux F.M., Mac-Thiong J.M., de Guise J.A., Berthonnaud E., Labelle H. (2006). Computerized assessment of sagittal curvatures of the spine: Comparison between Cobb and tangent circles techniques. J. Spinal Disord. Tech..

[67] Plaugher G., Cremata E.E., Phillips R.B. (1990). A retrospective consecutive case analysis of pretreatment and comparative static radiological parameters following chiropractic adjustments. J. Manip. Physiol. Ther..

[68] Polly D.W., Kilkelly F.X., McHale K.A., Asplund L.M., Mulligan M., Chang A.S. (1996). Measurement of lumbar lordosis. Evaluation of intraobserver, interobserver, and technique variability. Spine.

[69] Rastegar F., Contag A., Daniels A., Hiratzka J., Lin C., Chang J., Than K., Raslan A., Kong C., Nguyen N.L. (2018). Proximal junctional kyphosis: Inter- and intraobserver reliability of radiographic measurements in adult spinal deformity. Spine.

[70] Rehm J., Germann T., Akbar M., Pepke W., Kauczor H.U., Weber M.A., Spira D. (2017). 3D-modeling of the spine using EOS imaging system: Inter-reader reproducibility and reliability. PLoS ONE.

[71] Ruhinda E., Byanyima R.K., Mugerwa H. (2014). Reliability and validity of subjective assessment of lumbar lordosis in conventional radiography. East. Afr. Med. J..

[72] Russell B.S., Muhlenkamp-Wermert K.A., Hoiriis K.T. (2020). Measurement of lumbar lordosis: A comparison of 2 alternatives to the Cobb angle. J. Manip. Physiol. Ther..

[73] Segundo S.T., Valesin E.S., Filho Lenza M., Santos D.D., Rosemberg L.A., Ferretti M. (2016). Interobserver reproducibility of radiographic evaluation of lumbar spine instability. Einstein.

[74] Severijns P., Overbergh T., Thauvoye A., Baudewijns J., Monari D., Moke L., Desloovere K., Scheys L. (2020). A subject-specific method to measure dynamic spinal alignment in adult spinal deformity. Spine J..

[75] Suzuki H., Endo K., Mizuochi J., Kobayashi H., Tanaka H., Yamamoto K. (2010). Clasped position for measurement of sagittal spinal alignment. Eur. Spine J..

[76] Suzuki H., Imai N., Nozaki A., Hirano Y., Endo N. (2020). Anatomical sacral slope, a new pelvic parameter, is associated with lumbar lordosis and pelvic incidence in healthy Japanese women: A retrospective cross-sectional study. J. Orthop. Surg..

[77] Taghipour-Darzi M., Ebrahimi-Takamjani E., Salavati M., Mobini B., Zekavat H. (2009). Reliability of quality measures of movement in lumbar spine flexion-extension radiography. J. Back. Musculoskelet. Rehabil..

[78] Takahashi Y., Watanabe K., Okamoto M., Hatsushikano S., Hasegawa K., Endo N. (2021). Sacral incidence to pubis: A novel and alternative morphologic radiological parameter to pelvic incidence in assessing spinopelvic sagittal alignment. BMC Musculoskelet. Disord..

[79] Tallroth K., Ylikoski M., Landtman M., Santavirta S. (1994). Reliability of radiographical measurements of spondylolisthesis and extension-flexion radiographs of the lumbar spine. Eur. J. Radiol..

[80] Teyhen D.S., Flynn T.W., Bovik A.C., Abraham L.D. (2005). A new technique for digital fluoroscopic video assessment of sagittal plane lumbar spine motion. Spine.

[81] Timon S.J., Gardner M.J., Wanich T., Poynton A., Pigeon R., Widmann R.F., Rawlins B.A., Burke S.W. (2005). Not all spondylolisthesis grading instruments are reliable. Clin. Orthop. Relat. Res..

[82] Troyanovich S.J., Robertson G.A., Harrison D.D., Holland B. (1995). Intra- and interexaminer reliability of the chiropractic biophysics lateral lumbar radiographic mensuration procedure. J. Manip. Physiol. Ther..

[83] Troyanovich S.J., Harrison D.E., Harrison D.D., Holland B., Janik T.J. (1998). Further analysis of the reliability of the posterior tangent lateral lumbar radiographic mensuration procedure: Concurrent validity of computer-aided X-ray digitization. J. Manip. Physiol. Ther..

[84] Wang Z., Parent S., de Guise J.A., Labelle H. (2010). A variability study of computerized sagittal sacral radiologic measures. Spine.

[85] Wanke-Jellinek L., Heese O., Krenauer A., Würtinger C., Siepe C.J., Wiechert K., Mehren C. (2019). Is there any use? Validity of 4D rasterstereography compared to EOS 3D X-ray imaging in patients with degenerative disk disease. Eur. Spine J..

[86] Wong C., Hall J., Gosvig K. (2019). The effects of rotation on radiological parameters in the spine. Acta Radiol..

[87] Wu W., Liang J., Du Y., Tan X., Xiang X., Wang W., Ru N., Le J. (2014). Reliability and reproducibility analysis of the Cobb angle and assessing sagittal plane by computer-assisted and manual measurement tools. BMC Musculoskelet. Disord..

[88] Wu J., Wei F., Ma L., Li J., Zhang N., Tian W., Sun Y. (2021). Accuracy and reliability of standing lateral lumbar radiographs for measurements of spinopelvic parameters. Spine.

[89] Zhang Y., Hai Y., Liu Y., Zhang X., Zhang Y., Han C., Liu J., Zhou L. (2022). The reliability of computer-assisted three-dimensional surgical simulation of posterior osteotomies in thoracolumbar kyphosis secondary to ankylosing spondylitis patients. Mediat. Inflamm..

[90] Zhou Q.S., Sun X., Chen X., Xu L., Qian B.P., Zhu Z., Qiu Y. (2021). Utility of natural sitting lateral radiograph in the diagnosis of segmental instability for patients with degenerative lumbar spondylolisthesis. Clin. Orthop. Relat. Res..

[91] Zhou S., Yao H., Ma C., Chen X., Wang W., Ji H., He L., Luo M., Guo Y. (2022). Artificial intelligence X-ray measurement technology of anatomical parameters related to lumbosacral stability. Eur. J. Radiol..

[92] Zhu F., Bao H., He S., Wang F., Zhu Z., Liu Z., Qiu Y. (2015). Lumbo-femoral angle: A novel sagittal parameter related to quality of life in patients with adult scoliosis. Eur. Spine J..

[93] Lucas N.P., Macaskill P.M., Irwig L., Bogduk N. (2010). The development of a quality appraisal tool for studies of diagnostic reliability (QAREL). J. Clin. Epidemiol..

[94] Lopes M.A., Coleman R.R., Cremata E.J. (2021). Radiography and clinical decision-making in chiropractic. Dose Response.

[95] Traeger A., Buchbinder R., Harris I., Maher C. (2017). Diagnosis and management of low-back pain in primary care. CMAJ.

[96] Suits W.H. (2021). Clinical measures of pelvic tilt in physical therapy. Int. J. Sports Phys. Ther..

[97] Groisser B.N., Hillstrom H.J., Thakur A., Morse K.W., Cunningham M., Hresko M.T., Kimmel R., Wolf A., Widmann R.F. (2022). Reliability of automated topographic measurements for spine deformity. Spine Deform..

[98] Gold R., Esterberg E., Hollombe C., Arkind J., Vakarcs P.A., Tran H., Burdick T., Devoe J.E., Horberg M.A. (2016). Low back imaging when not indicated: A descriptive cross-system analysis. Perm. J..

[99] Dagenais S., Galloway E., Roffey D. (2014). A systematic review of diagnostic imaging use for low back pain in the United States. Spine J..

[100] Ghafouri M., Ghasemi E., Rostami M., Rouhifard M., Rezaei N., Nasserinejad M., Danandeh K., Nakhostin-Ansari A., Ghanbari A., Borghei A. (2023). The quality of care index for low back pain: A systematic analysis of the global burden of disease study 1990–2017. Arch. Public Health.

[101] Sylwander C., Larsson I., Andersson M., Bergman S. (2020). The impact of chronic widespread pain on health status and long-term health predictors: A general population cohort study. BMC Musculoskelet. Disord..

[102] Oakley P.A., Kallan S.Z., Harrison D.E. (2022). Structural rehabilitation of the lumbar lordosis: A selective review of CBP^®^ case reports. J. Contemp. Chiro.

[103] Harrison D.E., Oakley P.A. (2018). Non-operative correction of flat back syndrome using lumbar extension traction: A CBP^®^ case series of two. J. Phys. Ther. Sci..

[104] White H.J., Bradley J., Hadgis N., Wittke E., Piland B., Tuttle B., Erickson M., Horn M.E. (2020). Predicting patient-centered outcomes from spine surgery using risk assessment tools: A systematic review. Curr. Rev. Musculoskelet. Med..

[105] Kim G.U., Chang M.C., Kim T.U., Lee G.W. (2020). Diagnostic modality in spine disease: A review. Asian Spine J..

[106] Ames C.P., Smith J.S., Pellisé F., Kelly M., Alanay A., Acaroğlu E., Pérez-Grueso F.J.S., Kleinstück F., Obeid I., Vila-Casademunt A. (2019). Artificial intelligence based hierarchical clustering of patient types and intervention categories in adult spinal deformity surgery: Towards a new classification scheme that predicts quality and value. Spine.

[107] Hosseini M.M., Mahoor M.H., Haas J.W., Ferrantelli J.R., Dupuis A.-L., Jaeger J.O., Harrison D.E. (2024). Intra-examiner reliability and validity of sagittal cervical spine mensuration methods using deep convolutional neural networks. J. Clin. Med..

[108] Casiano V.E., Sarwan G., Dydyk A.M., Varacallo M. (2023). Back Pain.

[109] Manabe H., Morimoto M., Sugiura K., Takeuchi M., Tezuka F., Yamashita K., Sakai T., Sairyo K. (2024). Morphological evaluation of lumbar facet joints in professional baseball players. Orthop. J. Sports Med..

[110] Bortsov A.V., Parisien M., Khoury S., Martinsen A.E., Lie M.U., Heuch I., Hveem K., Zwart J.A., Winsvold B.S., Diatchenko L. (2022). Brain-specific genes contribute to chronic but not to acute back pain. Pain Rep..

[111] Vaedeh D., Mannion R.J., Woolf C.J. (2016). Toward a mechanism-based approach to pain diagnosis. J. Pain.

[112] Du S.H., Zhang Y.H., Yang Q.H., Wang Y.C., Fang Y., Wang X.Q. (2023). Spinal posture assessment and low back pain. EFORT Open Rev..

[113] Choi S., Nah S., Jang H.D., Moon J.E., Han S. (2021). Association between chronic low back pain and degree of stress: A nationwide cross-sectional study. Sci. Rep..

[114] Yang H., Lu M.L., Haldeman S., Swanson N. (2023). Psychosocial risk factors for low back pain in US workers: Data from the 2002–2018 quality of work life survey. Am. J. Ind. Med..

[115] Lerchi T., Nispel K., Baum T., Bodden J., Senner V., Kirschke J.S. (2023). Multibody models of the thoracolumbar spine: A review on applications, limitations, and challenges. Bioengineering.

[116] Pennington C.W., Siegel J.A. (2019). The linear no-threshold model of low-dose radiogenic cancer: A failed fiction. Dose Response.

[117] Oakley P.A., Harrison D.E. (2018). Radiophobia: 7 Reasons Why Radiography Used in Spine and Posture Rehabilitation Should Not Be Feared or Avoided. Dose Response..

[118] Schultz C.H., Fairley R., Murphy L.S., Doss M. (2020). The risk of cancer from CT scans and other sources of low-dose radiation: A critical appraisal of methodologic quality. Prehosp. Disaster Med..

[119] Selby P.B., Calabrese E.J. (2023). How self-interest and deception led to the adoption of the linear non-threshold dose response (LNT) model for cancer risk assessment. Sci. Total Environ..

[120] Calabrese E.J., Agathokleous E., Giordano J., Selby P.B. (2023). Manhattan Project genetic studies: Flawed research discredits LNT recommendations. Environ. Pollut..

[121] Calabrese E.J., Agathokleous E. (2022). Is LNT anti-evolution dose response model?. Arch. Toxicol..

[122] Doss M. (2018). The Conclusion of the BEIR VII Report Endorsing the Linear No-Threshold Model Is No Longer Valid Due to Advancement of Knowledge. J. Nucl. Med..

[123] Calabrese E.J., Giordano J. (2022). How did Hermann Muller publish a paper absent any data in the journal Science? Ethical questions and implications of Muller’s Nobel Prize. Chem. Biol. Interact..

[124] Calabrese E.J. (2023). Confirmation that Hermann Muller was dishonest in his Nobel Prize Lecture. Arch. Toxicol..

[125] Oakley P.A., Harrison D.E. (2019). Selective usage of medical practice data, misrepresentations, and omission of conflicting data to support the ‘red flag only’ agenda for chiropractic radiography guidelines: A critical assessment of the Jenkins et al. article: “Current evidence for spinal X-ray use in the chiropractic profession”. Ann. Vert. Sublux Res..

[126] Lee C.-H., Heo S.J., Park S.H., Jeong H.S., Kim S.-Y. (2019). Functional changes in patients and morphological changes in the lumbar intervertebral disc after applying lordotic curve-controlled traction: A double-blind randomized controlled study. Medicina.

