# Supplementary material for: Reliability of the Biomechanical Assessment of the Sagittal Lumbar Spine and Pelvis on Radiographs Used in Clinical Practice: A Systematic Review of the Literature
Source: J Clin Med. 2024 Aug 8;13(16):4650. doi: 10.3390/jcm13164650 (PMC11355792; doi:10.3390/jcm13164650)
Supplement: Supplementary file 1 [file jcm-13-04650-s001.zip › Table S1-Addendum-Link-StudyCharachteristics-MainTable.pdf]

Author/year	Sample#, age, sex	# Reviewers	Repeat analysis details	Type of Images	Method of analysis	Inter-rater reliability	Intra-rater reliability	SEM	BIAS RISK	QUALITY	LL	PI/PT (pelvic morphology)	SS	Intersegmental	sagittal regional translation
Abdel 2012 [29]	50, 18 yo+ (35 male, 15 female); Intra ob: 10 films	2	50 films assessed by two observers for inter-rater; 10 films assessed 2 times by each for intrarater; intervals not described	supine Xrays	segmental Cobb - ex/ L3Cobb = superior endplate L2 to Inferior endplate L4	avg ICC=.0902	avg ICC = 0.653	Not reported	L	H	0	0	0	1	
Ames 2015 [30]	10 patients with cervical spine deformity (age and sex undescribed)	20	20 experienced spinal deformity surgeons was queried to classify each case twice, with a minimum of 1 intervening week.	Xray	PT, PI-LL (LL: superior endplate of S1 and L1)	Fleiss k coefficients: (round 1/round 2) PT=[0.714], PI-LL=[0.554/0.386, 0.826]	(Fleiss k coefficients) PT =0.633, PI-LL=0.627	Not reported	M	M	1	1	0	0	0
Andreason 2007 [31]	22 (8 females, 14 males) avergae age 49.5 years (range, 27-69 years)	2	marked twice, 2 weeks between sets	Xray	LL: superior endplate of S1 and L1	Bland and Altman's limit of agreement (LOA) 95% CI=47 to 59 degrees	Bland and Altman's limit of agreement (LOA) 95% CI=47 to 59 degrees	Not reported	L	H	1	0	0	0	0
Bagheri 2018 [32]	15 patients with idiopathic scoliosis (averaged age: 11.9 years; range: 6-15 years)	2	marked twice, 2 days between tials	EOS	LL=L1-L5 endplate	ICC=0.94 (<.0001)	ICC=0.83 (<.0001)	less than 1 degree for LL	H	L	1	0	0	0	0
Bolesta 2010[33]	24	3	2 week interval	plain film (protractor) vs digital	LL=Superior L1-Superior L5	ICC (95% CI :)LL (digital 0.96 0.93–0.98, film 0.94 0.90–0.97)	ICC (95% CI :)LL (Exam 1 digital 0.97 0.93–0.99),(Examiner 1 film 0.96 0.91–0.98), Exam 2 digital 0.99 0.99–0.99, Exam 2 film 0.98 0.94–0.99), (exam 3 digital 0.98 0.94–0.99, Exam 3 Film 0.96 0.91–0.98)	Not reported	M	M	1	0	0	0	0
Bredow 2015 [34]	102	5	measured twice, one week interval	Xray	SS, LL=L1-S1 endplate	ICC of 0.944 in LL and 0.990 in SS	ICC for LL ranged from 0.966 to 0.992 , ICC for SS = 0.944 to 0.983	Not reported	M	M	1	0	1	0	0
Breen 2019 [35]	55 (22 females, 33 males) 38 yo +/-13.9 BMI 24 (for sagittal plane anlaysis)	2	6 week interval	fluoroscopy	Maximum intervertebral rotation (IV-RoM), maximum sagittal translation in fexion, sagittal disc height during fexion (maximal in neutral to minimal in fexion)	25	Not reported	Not reported	L	H	0	0	0	1	0
Cakir 2006 [36]	24 (14 females, 10 males) 40.2 yo +/- 5.3	3 observers	Obs 1:2 methods twice, min 8 week interval, Obs 2: 2 methods once, Obs 3: 2 methods once	flexion and extension radiographs	The segmental lordosis for L4–L5 was measured from the upper vertebral endplate of L4 to the lower vertebral endplate of L5; At L5–S1, the segmental lordosis was measured from the upper vertebral endplate of L5 to the superior endplate of S1	The "interobserver-intramethod" reliability: The reliability of different observers measuring with the same method: Cobb method (95% CI 7.4°/5.8°) was inferior to the super-imposition method (95% CI 4.9°/4.5°)	The "intraobserver-intramethod" reliability: The reliability of the same observer measuring with the same method: Obs 1=0.17° (0.617), Obs 2=0.04° (0.932)	Not reported	M	M	0	0	0	1	0
Chanplakorn 2011 [37]	100 (70 males, 30 females) age 33.3 ± 6.8 (21–50 years)	2	measured twice by two independent orthopedic trainees	xray	LL=T12 inferior endplate to S1, Pelvic Radius (PR in mm), PR-S1 angle, global PR-T12 angle, (3) regional lumbopelvic lordosis angles (PR-L2, PR-L4 and PR-L5 angles), sacral translation distance (HA-S1) and pelvic angle (PA).	Pearson correlation coefficients ranged from 0.9687 to 0.9952 with P value<0.0001.	Not reported	Not reported	H	L	1	1	0	0	0
Chen 1999 [38]	16 age 28.5 yo range 24-41	3	measures repeated 2 weeks later	xray	vertebral centroid measurement of lumbar lordosis L1-L5 (CLL), Cobb superior L1-inferior endplate L5, Cobb superior endplates L1-S1	Pearsons r (mean) Cobb L1-5=0.826; Cobb L1-S1=0.784; CLL=0.903	Pearsons r: Cobb L1-5=0.918-0.972; Cobb L1-S1=0.894-0.973; CLL=0.904-0.982	Intra Obs Mean Absolute Difference = Cobb L1-5=2.0 deg; Cobb L1-S1=2.2 deg; CLL=1.6 deg; Inter Obs Mean Absolute Difference = Cobb L1-5=4.0 deg; Cobb L1-S1=4.8 deg; CLL=1.3 deg	M	M	1	0	0	0	0
Chung 2017 [39]	50 66.3 years (range, 25–91 y) 18 males 32 females	3	measured 2 times by 3 observers	Digital Xray , lateral whole spine vs lateral lumbar	LL=Superior endplate L1-inferior endplate L5, SS, PI, PT	ICCs for LL and PT measured on lateral whole-spine radiographs (0.956–0.975 and 0.980–0.986), ICC's on lateral lumbar radiographs (0.948–0.964 and 0.945–0.970)	intraobserver ICCs were >0.75 (good-to-excellent)	Not reported	L	H	1	1	1	0	0
de Carvalho 2010 [40]	20 (no age gender given)	2	measured twice, no interval given	1 standing 1 sitting in auto seat	LL= Cobb superior endplates L1-S1	Bland Altman LOA: rater 1: LOA= 4.48° and −4.68° were found to be the limits of agreement, (t = −0.1951; df = 19; P = .8474). Rater 2: The LOA are 6.79° and −5.69°, paired t-test ((t19 = 0.7884; P = .4402)	Bland Altman LOA: 2 measurements made by rater 1 differed by a score of 0.1° ± 2.3°; paired t test of the 2 sets of measurements completed by rater 1 supports our conclusion that there is no statistically significant difference between them (t =−0.1951; df = 19; P = .8474); second rater, an average difference of 0.5° ± 2.2°. The limits of agreement, mean difference plus or minus 2 SDs, are 6.79° and −5.69°	Not reported	H	L	1	0	0	0	0
Dimar 2008 [41]	29 asymptomatic young adults	10	48 hours apart		"Cobb" lumbar, SS, PI, PT	inter-rater reliability for the manual measures also varied from a low Pearson's coefficient of -0.08 for the L4 incidence to a high of 0.71 for the sacral slope	Manual: fair to moderate intra-rater reliability among manual measures in determining end vertebrae with kappa coefficients ranging from 0.47 to 0.72. inter-rater reliability for the manual measures also varied from a low ICC of -0.02 (-0.04 to 0.03) for the L4 incidence to a high of 0.64 (0.61–0.67) for the sacral slope.	Not reported	M	M	1	1	1	0	0
du Rose 2016 [42]	10 males	2	6 week interval	fluoroscopy	Intervetebral ROM	ICC=0.94-0.99	ICC=0.96-0.99	Intra:0.23-0.54 degrees; Inter: 0.24-0.76 degrees	M	M	0	0	0	1	0
Fritz 2005 [43]	10 (intra rater analysis)		10-14 day interval	flexion extension xrays	White and Panjabi-intervertebral Cobb angles and translation adjjusting for magnification	not repoted	Intraclass correlation coefficients values for radiographic variables ranged from 0.84 to 0.99 for translation measures and 0.81 to 0.96 for rotation measures.	Not reported	L	H	0	0	0	1	0
Gilliam 1994 [44]	15 (9 men 6 women), age 23.9 (20-44)	2	interval not described	xray	Radiographic Sacral Angle (SS)	ICC=0.86	ICC=0.92 and 0.95	Not reported	M	M	0	0	1	0	0
Gladnick 2017 [45]	24 (5 male, 19 fem) age 29.2 (7–59)		2 reviewers, 2 different assessment methods 1 time each, 1 week interval	digital xray	Cobb LL PACS and Post-It® notes and a goniometer	PACS: ICC=0.961 (lumbar lordosis) Post-It®:ICC=0.979	Not reported	Not reported	L	H	1	0	0	0	0
Harrison 2001 [46]	30	3	1 week interval	plain films-digitized	LL(Centroid, Cobb T12-S1, Cobb L1-L5, Post Tangent, TRALL)	ICC: Global Centroid=1.00 (0.99-1.00), Cobb T12-S1=0.98 (0.97-0.99), Cobb L1-L5 =0.98 (0.97-0.99), Post Tangent L1-5 =0.98 (0.96-0.99), TRALL=0.99 (0.99-1.00), SS=0.99 (0.98-0.99), PT=1.00 (1.00-1.00), sagittal balance T12-S1=1.00 (1.00-1.00)	ICC: Global Centroid=1.00 (0.99-1.00), Cobb T12-S1=0.99 (0.99-1.00), Cobb L1-L5 =0.98 (0.98-1.00), Post Tangent L1-5 =0.99 (0.97-0.99), TRALL=1.00 (0.99-1.00), SS=0.99 (0.99-1.00), PT=1.00 (1.00-1.00), sagittal balance T12-S1=1.00 (1.00-1.00)	LL(Centroid=0.8-1.0 deg, Cobb T12-S1, Cobb L1-L5, Post Tangent, TRALL)	L	H	1	0	1	1	1
Hicks [47]	48 age 75.23 +/- 4.79 BMI 30.32 +/- 6.03	2	no experience, no training (PhD, PTs)	plain xray - hand drawn	Cobb Superior L1-Inferior L5	ICC LL is 0.98 (95% CI: 0.95, 0.99).	Not reported	SEM for LL=1.99 degrees, MDC = 3.90	M	M	1	0	0	0	0
Hohenhaus 2022 [48]	43 (18 male, 25 fem) 57 years (interquartile range - IQR 48 - 69)	4	4 reviewers marked films preoperatively and follow-up films postoperatively	Xray-lateral full spine	Cobb LL (superior endplate L1-S1), SS, PI, PT	overall (between 4 examiners): ICCs: LL 0.906, SS 0.872, PI 0.913, PT 0.970	None performed	Not reported	M	M	1	1	1	0	0
Hong 2010 [49]	90 (30 normal, 30 low- and 30 high-grade adult scoliosis) 61.2 yo	3	measured twice by each of three examiners with 2 weeks delay between first and second measurement		Cobb L1–S1, Cobb L1–L5, centroid, posterior tangent L1–S1, posterior tangent L1–L5 and TRALL	ICC's:Cobb L1–S1 0.90, Cobb L1–L5 0.91, centroid 0.85, posterior tangent L1–S1 0.84, posterior tangent L1–L5 0.91 and TRALL 0.82	ICC's:Cobb L1–S1 0.97, Cobb L1–L5 0.97, centroid 0.97, posterior tangent L1–S1 0.96, posterior tangent L1–L5 0.96 and TRALL 0.93	Mean Absolute Difference (Intra-obs):Cobb L1–S1 3.07, Cobb L1–L5 2.53, centroid 3.49, posterior tangent L1–S1 3.03, posterior tangent L1–L5 3.03 and TRALL 3.00; Mean Absolute Difference (Inter-obs):Cobb L1–S1 4.14, Cobb L1–L5 3.76, centroid 4.27, posterior tangent L1–S1 4.14, posterior tangent L1–L5 3.23 and TRALL 3.27	L	H	1	0	0	0	0
Jackson 1998 [50]	160 adults (group 1:50 healthy volunteeers, group 2: 50 degenerative lumbar disc disease, group 3: 30 scoliosis, group 4: 30 spondylolisthesis)	2	each films measured 2x with 2 observers	lateral 36" xrays	LL= 0.73-0.94, Pelvic radius= 0.84-0.97, Sacropelvic angle= 0.78-0.98, sacral inclination (not SS)= 0.39-0.79, sacropelvic translation= 0.30-0.98 [degenerative group lowest - others good-excellent]	Pearsons r (4 groups): LL= 0.73-0.91, Pelvic radius= 0.84-0.97, Sacropelvic angle= 0.78-0.98, sacral inclination (not SS)= 0.39-0.90, sacropelvic translation= 0.30-0.98 [degenerative group lowest - others good-excellent]	Pearsons r (Obs1,2 for 4 groups): LL= Obs1: 0.95-0.99, Obs2: 0.87-0.99, Pelvic radius= Obs1: 0.87-0.99, Obs2: 0.70-0.96, Sacropelvic angle= Obs1: 0.95-0.99, Obs2: 0.86-0.99 , sacral inclination (not SS)= Obs1: 0.94-0.99, Obs2: 0.43-0.96, sacropelvic translation= Obs1: 0.87-0.99, Obs2: 0.93-0.99 [degenerative and scoliosis group lowest for observer#2 - others good-excellent]	Not reported	M	M	1	0	0	0	0

Jackson 2000 [51]	40 radiogrpahs of 20 patients	2	two radiographs taken 1 to 4 weeks apart (average, 2 weeks). The 40 radiographs of the 20 patients were measured again later by the first observer, then independently measured two more times on different days by a second observer.	xray- 36 inch lateral	Pelvic Radius (PR), PRS1 (During, Legaye), Pelvic Angle (PA), sacral translation (HAS1 and HASP), Lumbopelvic lordosis(PRT12 and PRL1), Regional lumbopelvis lordosis (PRL2, PRL3, PRL4, PRL5), total lumbosacral lordosis (inferior endplateT12-superior endplate S1, superior endplate L1-superior endplate S1)	Pearson's linear regression coefficients all total lordosis measures (Cobba and PR) ranged from 0.94-0.98 for both observers	Pearson's linear regression coefficients all total lordosis measures (Cobba and PR) ranged from 0.94-0.98 for both observers	Not reported	M	M	1	1	0	0	0
Karabag 2022 [52]	26 (14 male 12 fem)	2	2 experienced surgeons 2Xs	lateral L5/S1	Pelvic Incidence (PI) and Pelvic Tilt (PT)	PI= 0.88, PT=0.89	PI= 0.92, PT=0.91	Not reported	H	L	0	1	0	0	0
Kepler 2015 [53]	26	5	5 spine surgeons, and 1 spine fellow, 1 month interval, altered order	digital radiogrpahy - standing neutral, flexion and extension	L4/5 spondy distance	The Kappa value for the interobserver analysis (six observers) of the four morphologic subgroups was 0.82. (95% confidence interval, 0.74–0.90)	Kappa value of 0.83 (range, 0.77–0.89)	Not reported	L	H	0	0	0	1	0
Khalsa 2018 [54]	20 w/ lumbosacral transitional vertebra	16	16 surgeons 2 week interval	36 inch lateral full spine films	Lumbar Lordosis (undefined), Pelvic Incidence, Pelvic Tilt	PI (0.42, 95% CI 0.26, 0.65) and fair for LL (0.67, 95% CI 0.51, 0.82) and PT (0.63, 95% CI 0.47, 0.81)	Pearson correlation coefficient showed excellent IAOR (LL 0.86, TPA 0.77, PI 0.78, PT 0.86).	Not reported	M	M	1	1	0	0	0
Kunkle 2017 [55]	30 total (10 for intraobserver analysis)	3 (junior level orthopedic resident, a senior level orthopedic resident, and a fellowship-trained spinal deformity surgeon)	each measured 30 radiographs at two separate time points—once with a smartphone protractor application, and then again with a standard wax pencil and handheld protractor. The first 10 radiographs were each re-measured by the three observers at two additional time points using the smartphone application, and then two additional times using a wax pencil and standard protractor. This provided each observer with six separate measurements of the 10 radiographs-three using the smartphone application, and three using the wax pencil and standard protractor.	36 inch lateral full spine films for LL and conded down lumbosacral for PI and PT	Lumbar lordosis (superior endplates L1 and S1), Pelvic Incidence, Pelvic Tilt	LL (smartphone)= 0.980-0.993, PI (smartphone)= 0.970-0.975, PT (smartphone)= 0.874-0.969; LL (protractor)= 0.865-0.974 , PI (protractor)= 0.863-0.941, PT (protractor)= 0.807-0.959	LL= 0.983, 0.986, 0.986 , PI= 0.984, 0.990, 0.966 , PT= 0.989, 0.997, 0.958	Not reported	M	M	1	1	0	0	0
Lafage 2015 [56]	50	5 (2 experienced surgeons, 3 novice fellows)	1 week interval	biplanar stereoradiographic full body - EOS, and low dose full spine	PI, PT, SS, LL (superior endplates L1-S1), PI-LL mismatch	ICCs: PI=0.909, PT=0.978, SS=0.877, LL=0.872, PI-LL=0.872	ICCs: PI=0.956, PT=0.978, SS=0.943, LL=0.935, PI-LL=0.964	Not reported	L	H	1	1	1	0	0
Lee 2013 [57]	91 subjects, 17 women and 74 w/ ankyplosing spondylitis men, age 44.3 ± 13.5 years, ankylosing spondytis (39 non-ankylosis, 27 incomplete ankylosis and 25 complete ankylosis)	3	2 week interval	72 in, full spine, digital	6 methods LL: Centroid L1-S1, Centroid L1-L5, Centroid, Posterior tangent L1-S1, Posterior tangent L1-L5, TRALL	Centroid L1-S1=0.95, Centroid L1-L5=0.96, Centroid=0.86, Posterior tangent L1-S1=0.93, Posterior tangent L1-L5=0.90, TRALL=0.83	Centroid L1-S1=0.96, Centroid L1-L5=0.97, Centroid=0.90, Posterior tangent L1-S1=0.90, Posterior tangent L1-L5=0.85, TRALL=0.77	Not reported	H	L	1	0	0	0	0
Lee 2019 [58]	30 (17 fem, 13 males) age was 50.86 17.63 years (range, 20e78 years).	3 surgeons marked twice 1 week interval	Not Described	lateral full spione films	LL(upper endplates L1 and S1), SS, PI, PT	ICCs (PACS): LL=0.93, SS=0.89, PI=0.86, PT=0.90; ICCs (APP): LL=0.93, SS=0.87, PI=0.84, PT=0.93	ICCs (PACS): LL=0.98, SS=0.97, PI=0.98, PT=0.97 ;ICCs (APP): LL=0.96, SS=0.92, PI=0.95, PT=0.93	Not reported	M	M	1	1	1	0	0
Marchetti 2017 [59]	90 ((48 girls and 42 boys), average age 12.1 ± 4.9 years)	3	1 week interval	digital lateral full spine	Cobb L1-L5	ICC Cobb L1-L5=0.765 (0.695-0.827)	ICC Cobb L1-L5=0.878 (0.764-0.891)	Intra=3.8 deg, Inter=4.3 deg	L	H	1	0	0	0	0
McCarty 2009 [60]	30	4	2 days -2 weeks	digital Lateral lumbar radiogrpahs	6-line method resulting in 4 measures of slip%	4 methods ranged from 0.59-0.85	4 methods ranged from 0.69-0.87	Not reported	M	M	0	0	0	1	0
Mellor 2014 [61]	10	2	not described	lateral lumbar flexion and extension motions- video fluoroscopy	intersegmental ROM according to Frobin L2/3, L3/4, L4/5	Flexion L2/3=0.912, L3/4=0.975, L4/5=0.967; Extension L2/3=0.761, L3/4=0.737, L4/5=0.988	Flexion L2/3=0.959, L3/4=0/981, L4/5=0.997; Extension L2/3=0.959, L3/4=0.920, L4/5=0.993	0.125 degrees-0.988 degrees	M	M	0	0	0	1	0
Newton 2016 [62]	60 (ages 10-21) scoliosis	2	"another day"	2D EOS and 3D EOS	SS, PI, PT	Interrater ICC: SS=0953-0.97, PI=0.951-0.977, PT= 0.966-0.988	Intrarater ICC: SS=0.958- 0.98, PI=0.958-0.987, PT= 0.978-0.999	Not reported	M	M	0	1	1	0	0
Okpala 2018 [63]	200 (100 males, 100 females; 16-72 yo)		not described	plain Xrays - protractor and ruler	TRALL, LL Cobb L1-S1, Lumbosacral angle (LSA=SS), Luombosacral joint angle-JSJA (Inferior endplate L5 to Superior angle S1)	only SDs reported LSJA=5.7, TRALL=8.3, LSA=10.0, Cobb=12.8	Not reported	Not reported	H	L	1	0	1	0	0
Orosz 2022 [64]	100 patients (200 films - pre and post surgery)	2	not described	digital DICOM	Artifical Intelligence (convolutional neural network) vs PACS by 2 human (Raters 1 and 2): LL, PI, PT, and SS	PRE-OP: LL (ICC rater 1 vs 2=0.92 (0.86 to 0.95), Pearson R=0.92; PI (ICC rater 1 vs 2=0.96 (0.92 to 0.97), Pearson R=0.96; PT (ICC rater 1 vs 2=0.93 (0.88 to 0.96), Pearsons R=0.93 and SS (ICC rater 1 vs 2)=0.92 (0.86 to 0.95), Pearsons R=0.92 POST-OP: LL (ICC rater 1 vs 2=0.94 (0.90 to 0.97), Pearson R=0.94; PI (ICC rater 1 vs 2=0.95 (0.91 to 0.97), Pearson R=0.96; PT (ICC rater 1 vs 2=0.94 (0.89 to 0.97), Pearsons R=0.95 and SS (ICC rater 1 vs 2)=0.90 (0.83 to 0.94), Pearsons R=0.91	Not reported	Root Mean Square Error: PRE-OP (rater 1 vs 2): LL=2.7 deg ; PI=3.4 deg; PT=2.3 deg and SS=2.6 deg POST-OP: (rater 1 vs 2): LL=2.7 deg ; PI=2.7 deg; PT=1.5 deg and SS=2.6 deg	M	M	1	1	1	0	0
Pearson 2011 [65]	30 (66yo 63% female)	3	not described	plain films and computer assisted digital	intersegmental rotation and translation	The ICC(3,1)s for the digitized manual measurements ranged from 0.484 (L1-L2) to 0.780 (L3-L4) for intervertebral rotation (0.693 overall), 0.122 (L1-L2) to 0.184 (L5-S1) for AP translation (0.151 overall), 0.044 (L2-L3) to 0.408 (L4-L5) for anterior disc height change (0.373 overall), and 0.050 (L5-S1) to 0.446 (L3-L4) for posterior disc height change (0.300 overall). The confidence intervals included zero at all intervertebral levels for AP translation and at multiple levels for anterior and posterior disc height change. In contrast, the ICC(3,1)s for the QMA measurements ranged from 0.912 (L5-S1) to 0.962 (L4-L5) for intervertebral rotation (0.976 overall), 0.343 (L5-S1) to 0.864 (L3-L4) for AP translation (0.862 overall), 0.709 (L5-S1) to 0.839 (L4-L5) for anterior disc height change (0.869 overall), and 0.668 (L1-L2) to 0.808 (L2-L3) for posterior disc height change (0.730 overall).	measurements were observed for intervertebral rotation. The ICC(3,1)s for rotation ranged from 0.708 (L1-L2) to 0.866 (L4-L5) for the digitized manual measurements (0.870 overall) compared with 0.962 (L5-S1) to 0.990 (L3-L4) for QMA measurements (0.997 overall). The ICC(3,1)s were markedly lower for the digitized manual translational measurements. The ICC(3,1)s for the digitized manual measurements ranged from 0.083 (L3-L4) to 0.671 (L2-L3) for AP translation (0.557 overall), 0.234 (L1-L2) to 0.849 (L4-L5) for anterior disc height change (0.770 over- all), and 0.072 (L5-S1) to 0.628 (L3-L4) for posterior disc height change (0.283 overall).	Not reported	L	H	0	0	0	1	0
Pinel-Giroux 2006 [66]	10 controls (14.6 yo 6 females), 10 AIS (15.1 yo), 2 fem, 10 spondy (14.2 yo 6 fem)	3	4-6 week interval	lateral ful spine	LL Method 1: Max Cobb= suerior endplate S1-superior emdplate mot tilted vertebrae (e.g. T12, Method 2: Tangent Circles (endplates)	ICC LL Max Cobb= 0.97, TC=0.88	ICC LL Max Cobb= 0.77, TC=0.94	Not reported	M	M	1	0	0	0	0
Plaugher 1990 [67]	100 subjects (200 films-pre and post)	Inter: 3 examiners; intra 1 examiner		plain films	hand measured: L5 retrolisthesis	Pearsons R: L5 retrolisthesis (Obs A and B=0.74, A and C=0.79 B and C=0.83)	Pearsons R Observer A = L5 retrolisthesis 0.90)	L5 listhesis Inter=0.82mm, 0.72mm, 0.67mm	M	M	0	0	0	1	0
Polly 1996 [68]	60 (38 fem, 22 male, 13 healthy controls, 47 various conditions)	3 (orthopedic surgeon, pediatric orhtopedist, skeletal radiologist)	2 week interval, blinded, re-ordered	plain Xray lateral full spine	LL 4 Cobb methods: inferior endplate T12-superior endplate S1, superior endplate L1-superior endplate S1, inferior endplate T12-inferior endplate L5, superior endplate L1-inferior endplate L5	ICCs: ranged from 0.81-0.92	ICCs: ranged from 0.83-0.92	Not reported	L	H	1	0	0	0	0
Rastegar 2018 [69]	52 (>18 YO)	9	4 weeks later	digital lateral full psine films	TLJ kyphosis (Cobb)	Combined 9 eviewers TL ICC measure 1=0.965 measure 2=0.975	Range 9 Reviewers TL measure ICC mean=0.898 (0.817–0.969)	Not reported	L	H	0	0	0	0	0
Rehm 2017 [70]	73 (31 males, 42 fem)	2	not described	biplaner stereoradiography EOS	PI, SS, L1-5 Cobb, L1-S1 Cobb	ICCs: PI=0.97, SS=0.96, L1-5 Cobb=0.90, L1-S1 Cobb=0.85	Not reported	Not reported	H	L	1	1	1	0	0
Ruhinda 2014 [71]	70 (28 males, 42 fem, 18-70 yo)	2	not described		4 LL: vertebral centroid, TRALL, L1-5 posterior tangent, Cobb inf L1-sup S1)	only reported pooled results: kappa 0.36 (1st assess), 0.16 (2nd assess)	only reported pooled results: kappa 0.226 (1st examiner), 0.542 (2nd examiner)	Not reported	H	L	1	0	0	0	0

Russell 2020 [72]	16 (8 fem, 8 male, age 32.6, 21-61	2	not descrbed	lateral lumbar digital	2 LL methods: Cobb L1-L5, Harrison posterior tangent L1-L5	ICC Cobb 0.952, Post tang=0.985	ICC: Cobb: ex 1=0.968, ex 2=0.983, Post tang ex 1=0.991, ex 2=0.989	Not reported	M	M	1	0	0	0	0
Segundo 2016 [73]	126	2	The images were evaluated and classified by both observers.	Lateral lumbosacral spine radiographs in maximal flexion and extension..	Measurements were made of the dynamic lumbar spine radiographs in side views, assessing L4/5 and L5/S1 anterior translation and angulation between the vertebral bodies.	Proportion of Agreement (PA): L4-L5: Sagittal instability = 0.90, Angular instability = 0.93, instability based on endplates = 0.94 L5-S1: Sagittal instability = 0.90, Angular instability = 0.88, instability based on endplates = 0.94 Kappa: L4-L5: Sagittal instability = 0.43 (0.28-0.59), Angular instability = 0.57 (0.40-0.74), instability based on endplates = -0.03 (-0.21-0.14) L5-S1: Sagittal instability = 0.32 (0.15-0.50), Angular instability = 0.35 (0.21-0.50), instability based on endplates = -0.03 (-0.20-0.14) Agreement coefficient (AC1) L4-L5: Sagittal instability = 0.87 (0.80-0.95), Angular instability = 0.91 (0.86-0.97), instability based on endplates = 0.93 (0.88-0.98) L5-S1: Sagittal instability = 0.88 (0.81-0.95), Angular instability = 0.86 (0.78-0.93), instability based on endplates = 0.94 (0.90-0.99)	Not reported	Not reported	M	M	0	0	0	1	0
Severijns 2020[74]	8 ASD patients, 8 controls	3	The interval between sessions was maximum 2 weeks, to avoid changes in clinical status or deformity progression.	Standing lateral EOS® radiography	Lumbar Lordosis T12-S1 (LL)	Control: LL = 0.93 (0.80-0.99), ASD: LL = 0.79 (0.48-0.95)	The intrarater results for radiographic parameters and polynomial with correction, both during stance and STS, showed almost perfect ICCs with very small SEMs. Control: LL = 0.78 (0.29-0.95), ASD: LL =0.94 (0.75-0.99)	Inter-rater reliability: Control: LL = 2.80, ASD: LL = 8.85 Intrarater reliability: Control: LL = 4.97, ASD: LL = 4.47	M	M	1	0	0	0	0
Suzuki 2010 [75]	144 women	2	Measured twice with 1-week interval	standing radiography of the lumbar spine	PI: pelvic incidence; SS: sacral slope; PT: pelvic tilt; a-SS: anatomical sacral slope; LL: lumbar lordosis.	PI = 0.653, SS = 0.866, PT = 0.674, a-SS = 0.856, LL = 0.703	PI = 0.840, SS = 0.896, PT = 0.821, a-SS = 0.884, LL = 0.840	Not reported	M	M	1	1	1	0	0
Suzuki 2020 [76]	48 total: 26 adult volunteers, 15 males & 11 females, 32.7 +/- 8.3 y; 22 patients with lumbar spinal canal stenosis (LSCS), 12 males & 10 females, 62.0 +/- 15.4 y	2	Two radiographs were taken 1–4 weeks apart. The radiographs were measured twice by the first observer then independently measured at other days by a second observer.	Lateral long whole-spine radiography (clasped position).	Sagittal vertical axis (SVA), lumbar lordotic angle (LLA), pelvic angle (PA), pelvic lordosis angle (PRS1), pelvic tilt (PT), and pelvic incidence (PI).	The ICC value of SVA, LLA, PA, PRS1, PT, and PI were as follows: interrater reliability of volunteers 0.95 (95% CI 0.90–0.98), 0.97 (95% CI 0.94–0.99), 0.93 (95% CI 0.85–0.97), 0.98 (95% CI 0.96–0.99), 0.97 (95% CI 0.88–0.97), and 0.96 (95% CI 0.92–0.98), respectively. The interobserver agreement rates with measurements in clasped position were high, i.e., all results showed 'almost perfect agreement'.	The ICC value of SVA, LLA, PA, PRS1, PT, and PI were as follows: intrarater reliability of normal subjects 0.84 (95% CI 0.68–0.92), 0.92 (95% CI 0.84–0.96), 0.89 (95% CI 0.77–0.95), 0.92 (95% CI 0.83–0.96), 0.97 (95% CI 0.94–0.99), and 0.97 (95% CI 0.93–0.99), respectively. The ICC value of SVA, LLA, PA, PRS1, PT, and PI were as follows: intrarater reliability of patients 0.86 (95% CI 0.64–0.97), 0.91 (95% CI 0.80–0.96), 0.86 (95% CI 0.78–0.90), 0.90 (95% CI 0.77–0.96), 0.93 (95% CI 0.85–0.97), and 0.94 (95% CI 0.86–0.98), respectively.	Not reported	M	M	1	1	0	0	1
Taghipour-Darzi 2009 [77]	15 males, mean age 31.4 +/- 7.2 y (22-43 y)	1	Estimation of variables was done on two occasions, one week apart.	5 standing Lateral lumbar X-rays in neutral, full extension, full flexion, mid extension, and mid flexion positions were taken.	Center of rotation (COR) and centroid length of 3 artcs of vertebral movement containing full arc, flexion arc, and extension arc.	not repoted	Full arc COR for most lumbar segments have high ICC values on x axis (range: 0.67–0.88) and y axis (range: 0.55–0.90). At y axis, L1–2 segment had highest and L5-S1 segment had lowest ICC value equal to 0.90 and 0.55 respectively. ICC values of flexion arc COR in most lumbar segments were high on x axis (range: 0.54–0.97) and y axis (range: 0.48–0.99). Among lumbar segments, L2–3 had highest ICC at x and y axis equal to 0.97 and 0.99 respectively. However, L5–S1 segment had lowest ICC in estimation of flexion COR at x and y axis equal to 0.54 and 0.48 respectively. In comparison with two above measures, calculation of extension arc COR and centroid length had low ICC. ICC values of extension arc ranged between 0.19–0.60 in x axis with highest value for L2–3 segment and lowest for L3–4 segment. ICC of extension arc COR in y axis had range between 0.16–0.83 that highest value was for L2–3 level and lowest was for L3–4 level. ICC values for centroid length ranged between 0.22–0.88. For this variable, L1–2 level had highest ICC and L5–S1 level had lowest ICC.	The SEM values of full arc COR in most lumbar segments were low at x axis (range: 1.97–5.19) and y axis (range: 2.83–9.48). Estimation of COR of flexion arc had low SEM in x axis (range: 3.47–9.50) and y axis (range: 3.18–9.12). Extension arc COR and centroid length had high SEM for most lumbar segments. The SEM values ranged between 13.17–85.99 in x axis and 23.68–51.65 in y axis. Centroid length SEM values ranged between 97.31–218.59.	M	M	0	0	0	0	0
Takahashi 2021 [78]	30, age 20-70 y	3	3 authors independently and blindly measured 30 cases selected randomly from the 100 patients to compare inter-rater reliability. In addition, these measurements were repeated by the same three authors after more than 2 weeks to compare intra-rater reliability.	Standing slot scanning x-ray image	Pelvic Incidence (PI), Sacral incidence to pubis (SIP), Sacral slope (SS), Pelvic Tilt (PT), Pelvic incidence angle (PIA), Lumbar lordosis (LL)	Reliability analysis showed high inter-rater agreements in all the spinopelvic parameters, with ICCs > 0.9.ICC: PI = 0.98 (0.95-0.99); SIP = 0.96 (0.96-0.98); SS = 0.95 (0.91-0.98); PT = 0.99 (0.94-1.00); PIA = 0.99 (0.98-1.00); ll = 0.96 (0.92-0.98)	Reliability analysis showed high intrarater agreements in all the spinopelvic parameters, with ICCs > 0.9. ICC: Examinar 1: PI = 0.97 (0.94-0.99); SIP = 0.96 (0.92-0.98); SS = 0.95 (0.89-0.97); PT = 0.99 (0.98-1.00); PIA = 0.99 (0.99-1.00); ll = 0.96 (0.92-0.98) Examinar 2: PI = 0.98 (0.97-0.99); SIP = 0.98 (0.96-0.99); SS = 0.98 (0.95-0.99); PT = 1.00 (0.99-1.00); PIA = 0.99 (0.99-1.00); ll = 0.98 (0.96-0.99) Examinar 3: PI = 0.99 (0.99-1.00); SIP = 0.99 (0.98-1.00); SS = 0.99 (0.98-1.00); PT = 1.00 (0.99-1.00); PIA = 0.99 (0.99-1.00); ll = 0.99 (0.99-1.00)	Not reported	L	H	1	1	1	0	0
Tallroth 1994 [79]	30 (18 male, 12 female), mean age of 34 +/- 9 y (13-51 y)	3	The radiographs were evaluated in a blinded manner by two Each set of radiographs was assessed by the same observer on two occasions separated by at least 2 weeks.	Lateral lumbar flexion (seated), and extension (standing)	Angular movement (i.e. the total extension-flexion range) of L3 to S1 was measured. Intervertebral sagittal translatory movements were measured by calculating the position of the posterior inferior lip of the upper vertebral body in relation to the line along the upper surface of the inferior vertebral body. Posterior movement of the superior vertebra was indicated as a negative value.	The mean interobserver segmental angular variation (Reader 1 vs. 2; 1 vs. 3 and 2 vs. 3) at the L3-L4 level was 1.9° (SD 1.4°), at L4-L5 2.3° (SD 1.9°) and at the L5-S1 level 2.6° (SD 2.3°). In the sagittal translation, the mean interobserver variation at the L3-L4 level was 1.3 mm (SD 1.1 mm), at L4-L5 1.4 mm (SD 1.2 mm) and at the L5-S1 level 1.3 mm (SD 1.4 mm). At the neutral position, the mean interobserver variation in the L5 translation was 1.3 mm (SD 1.1 mm). The highest angular interobserver variation was found at the L5-S1 level (mean 2.6°, SD 2.3°, max 11°) while the highest variation in sagittal translatory movement was found at the L4-L5 level (mean 1.4 mm, SD 1.2 mm, max 6 mm). The mean interobserver variation for L5 olisthesis was 1.3 mm (SD 1.1 mm, max 6 mm). The overall consistency and concordance were good.	The mean intraobserver angular variation was at the L3-L4 level 1.4° (SD 1.3°), at L4-L5 1.5° (SD 1.3°) and at L5-S1 1.6° (SD 1.6"). The mean variation in sagittal translation was at the L3-L4 level 0.7 mm (SD 0.7 mm), at L4-L 50.7 mm (SD 0.7 mm) and at the L5-S1 level 0.6 mm (SD 0.8 mm). At the neutral position, the mean variation in the L5 translation was 1.0 mm (SD 0.9 mm). The highest intraobserver angular variations were found at the LS-S1 level (mean 1.6°, SD 1.6°, max 9°) and the highest sagittal translation also at the LS-S1 level (mean 0.6 mm, SD 0.8 mm, max 4 mm). The mean intraobserver variation for L5 olisthesis was 1.0 mm (SD 0.9 mm, max 5 mm). The overall consistency and concordance were good.	Not reported	L	H	0	0	0	1	0
Teyhen 2005 [80]	20 males, 11 with LBP (36.4 +/- 7.2 y [24-45 y]) and 9 control without LBP (30.4 +/- 8.0 y [19-44 y]) 40 images (20 lumbar flexion, 20 upright)	1	Repeated measures of 20 subjects were analyzed in the upright and flexed postures (3 repetitions of 40 single images, 240 analyzed images total, 3360 individually placed points). Interimage reliability assessed the reliability of data obtained from two separate movement trials, separated by a 2-minute rest and 2-minute walk break. To minimize rater bias the analysis of the first and second movement trials by the rater were separated by a minimum of 2 months.	Digital fluoroscopic video (DFV)	intervertebral angle between adjacent midplane lines (MPL); intersegmental displacement; L3-S1 lordosis angle (LA)between the MPL of L3 and the cephalad border of S1.	not repoted	The intraimage reliability for intersegmental angle and displacement range, ICC was between 0.96 and 0.99. The SEM ranged from 0.4 to 0.7° and 0.57 to 0.89% displacement (0.2–0.3 mm). The average interimage reliability, ICC, for minimum and maximum intersegmental angle was 0.91 (0.82–0.94), and displacement was 0.84 (0.64–0.93). The SEM ranged from 0.7 to 1.4° and 1.2 to 2.1% displacement (0.4–0.7 mm). The average SEM across all segments was 1° and 0.6 mm	SEM ranged from 0.7–1.4° and 0.4 –0.7 mm. The use of DCRA to measure the kinematic variables of the lumbar spine was a reliable technique with an average interimage SEM 2° and 1.2 mm (95% CI).	M	M	1	0	0	1	0
Timon 2005 [81]	30	4	Two attending orthopaedic spine surgeons and two orthopaedic spinal surgery fellows with less than 6 months experience. Six weeks interval	Standing lateral lumbar radiographs - hand measured	Spondylolisthesis grading instruments: 1) slip percentage, 2) Meyerding's grade, 3) slip angle, 4) lumbosacral angle, 5) angle of kyphosis, 6) lumbar index, 7) sagittal rotation, and 8) sacral inclination for each radiograph.	3 of the measurements had an interobserver correlation in the excellent range (slip percent, 0.89; Meyerding's grade, 0.78; sacral inclination, 0.82). The Meyerding's grade had a percentage agreement of 73% (22/30 patients). The lumbosacral angle had the lowest interobserver reliability (K = 0.08), and was the only scale in the poor correlation range. 1) slip percentage = 0.89 (0.83-0.94), 2) Meyerding's grade = 0.78** (0.66-0.86), 3) slip angle = 0.57 (0.43-0.72), 4) lumbosacral angle = 0.08 (0.00-0.21), 5) angle of kyphosis = 0.45 (0.31-0.62), 6) lumbar index = 0.41 (0.27-0.58), 7) sagittal rotation = 0.60 (0.46-0.74), and 8) sacral inclination = 0.82 (0.73-0.90) **Kappa correlations were used for Meyerding's grade; ICCs were used for the remaining scales	The 4 raters had mean ICCs of at least 0.76 (range, 0.76–0.91). The two attending orthopaedic surgeons (RFW and BAR) had individual intraobserver reliabilities of 0.80 and 0.76, and the two fellows had intraobserver reliabilities of 0.85 and 0.91. For intraobserver reliability of each of the measurements, 6/8 had correlations greater than 0.75 (slip percent, Meyerding's grade, slip angle, lumbosacral angle, sagittal rotation, and sacral inclination). The kyphosis angle (K = 0.73) and lumbar index (K = 0.51) were considered fair to good 1) slip percentage = 0.94 (0.88-0.97), 2) Meyerding's grade = 0.79** (0.50-0.91), 3) slip angle = 0.88 (0.77-0.94), 4) lumbosacral angle = 0.89 (0.80-0.94), 5) angle of kyphosis = 0.76 (0.56-0.85), 6) lumbar index = 0.51 (0.18-0.73), 7) sagittal rotation = 0.92 (0.85-0.96), and 8) sacral inclination = 0.84 (0.70-0.92) **Kappa correlations were used for Meyerding's grade; ICCs were used for the remaining scales	Not reported	M	M	0	0	0	1	0
Troyanovich 1995 [82]	35	3	A blind, repeated-measures design was used. Lateral lumbopelvic radiographs were presented to each of three examiners in random order. Each film was marked and measurements were recorded. The films were cleaned of all markings and randomized again for a second run by each examiner. Each examiner's measurements were unavailable to the other examiners.	Lateral lumbar radiographs	Anterior/posterior thoracic translation in millimeters, Ferguson's sacral-plane angle to horizontal, arcuate line angle to horizontal, L1 to L5 absolute rotation angle and four relative rotation angles for L1-L2, L2-L3, L3-L4 and L4-L5. Intra- and interrelibility of the three radiographic examiners were analyzed.	Interexaminer reliabilities for the three examiners ranged from .66-.98.	Intraexaminer reliability for (a) L1-L5 absolute rotation angle was .98, with confidence intervals included in the range of 0.95-0.99, (b) anterior/posterior thorax translation [+/- Sz] was .97-.99, with confidence intervals included in the range of 0.94-1.00, (c) arcuate angle (AA) .40-.81, with confidence intervals included in the range of 0.07-0.90, (d) Ferguson's angle (FA) was .91-.97, with confidence intervals included in the range of 0.82-0.98, (e) relative rotation angle reliability ranges were L1-L2, .84-.94; L2-L3, .80-.85; L3-L4, .78-.89; L4-L5, .87-.92.	Not reported	L	H	1	1	1	1	1

Troyanovich 1998 [83]	50	3	A blind, repeated-measures design was used. The results of radiographic measures derived through the traditional manual marking method were compared with measures derived by computer-aided digitization of lateral lumbopelvic radiographs.	Lateral lumbar radiographs	Relative rotation angles for TI2-LI, LI-L2, L2-L3, L3-L4, L4-L5, L5-S 1, overall lordosis measurement [absolute rotation angle (ARA)] from LI-L5 and Cobb angle of overall lordosis measured from the inferior surface of T12 to the superior surface of S1, Ferguson's sacral base angle to horizontal, angle of pelvic tilt (arcuate angle) to horizontal and anteroposterior thoracic translation (S(z)) in millimeters.	Average ICC of interexaminer reliability for manual and computer-aided digitizing examiners were the following: 0.96 for the L1-L5 ARA; 0.84 for the arcuate angle measurement; 0.82 for the Ferguson's angle measurement; 0.88 for the Cobb angle measurement; 1.00 for the S(z) translation measurement; and values of 0.65, 0.73, 0.74, 0.75, 0.89 and 0.81 for relative rotation angle measurements TI2-LI, L1-L2, L2-L3, L3-L4, L4-L5 and L5-S1, respectively.	ICC estimates for intraexaminer reliability were in the range of 0.96-0.98 for the L1-L5 ARA, a range of 0.87-0.99 for the arcuate angle measurement, 0.83-0.94 for the Ferguson's angle measurement, 0.88-0.95 for the Cobb angle measurement from the inferior surface of T12 compared with the superior surface of S1 and 0.98-1.00 for the translation measurement of the lower thoracic spine to S1 (S(z)). The intersegmental measurement's (T12-L1, L I-L2, L2-L3, L3-L4, L4-L5, L5-S 1) correlations ranged from a low of 0.55 to a high of 0.97. Examination of these findings suggests that the reliability for the three doctors is acceptable with only the TI2-LI intersegmental measure falling below 0.70 for the least experienced examiner.	Not reported	L	H	1	0	1	1	1
Wang 2010 [84]	30 (10 males, 20 females, 10-17 y). Three groups of 10 radiographs (10 radiographs of asymptomatic individuals, 10 of subjects with low grade L5–S1 spondylolisthesis, and 10 with high grade L5–S1 developmental spondylolisthesis)	3	Three surgeons measured sacral morphologic variables on the 30 radiographs at 2 occasions, with a 15 days interval between the 2 sessions, using a computer assisted technique.	Standing lateral radiographs of 30 to 90 cm - clavicular fossa position	1) STA, angle between a line along the sacral endplate and a line drawn along the posterior aspect of S1; 2) S1 superior (S1), angle between the median of S1 and the line perpendicular to the middle of the upper endplate of S1; 3) S2 inferior (S2), angle between the perpendicular of the middle of the lower endplate of S2 and the median of S1; 4) Sacral Kyphosis Ferguson (SKF), angle between a line joining the midpoint of the superior and inferior borders of S1 and the line joining the midpoint of the inferior borders of S2 and S4; 5) Sacral kyphosis Cobb (SKC) using the superior endplate of S1 and inferior endplate of S4.	ICC for all subjects (Asymptomatic, Low Grade and High Grade Groups Altogether): STA = 0.966 (0.945-0.986), S1 superior = 0.987 (0.981-0.993), S2 inferior = 0.949 (0.906-0.992), SKF = 0.995 (0.989-1.000), SKC = 0.957 (0.931-0.983) ICC for Asymptomatic subjects: STA = 0.975, S1 superior = 0.989, S2 inferior = 0.984, SKF = 0.997, SKC = 0.985 ICC for Low Grade L5-S1 Spondylolisthesis subjects: STA = 0.981, S1 superior = 0.990, S2 inferior = 0.9505, SKF = 0.995, SKC = 0.9505 ICC for High Grade L5-S1 Spondylolisthesis subjects: STA = 0.941, S1 superior = 0.982, S2 inferior = 0.913, SKF = 0.991, SKC = 0.9355	ICC for all subjects (Asymptomatic, Low Grade and High Grade Groups Altogether): STA = 0.951 (0.939-0.962), S1 superior = 0.991 (0.989-0.993), S2 inferior = 0.963 (0.949-0.981), SKF = 0.995 (0.990-1.001), SKC = 0.967 (0.956-0.978) ICC for Asymptomatic subjects: STA = 0.978, S1 superior = 0.991, S2 inferior = 0.987, SKF = 0.993, SKC = 0.987 ICC for Low Grade L5-S1 Spondylolisthesis subjects: STA = 0.984, S1 superior = 0.990, S2 inferior = 0.946, SKF = 0.997, SKC = 0.946 ICC for High Grade L5-S1 Spondylolisthesis subjects: STA = 0.889, S1 superior = 0.991, S2 inferior = 0.954, SKF = 0.992, SKC = 0.9666	Not reported	M	M	0	1	0	0	0
Wanke-Jellinek 2019 [85]	34 individuals (22 female, 12 male, 25-88 y)	4	Biplanar low-dose EOS images were analyzed using the sterEOS software (EOS imaging, Paris, France). To evaluate the interobserver reliability of the 3D EOS analysis, four equally experienced investigators separately analyzed each 3D EOS image using the sterEOS software.	Biplanar EOS X-ray images	EOS X-ray images were analyzed using the sterEOS software to determine the lumbar lordosis (LL).	ICC for LL was excellent (ICC = 0.9 [0.84; 0.94 95% CI])	Not reported	Not reported	H	L	1	0	0	0	0
Wong 2019 [86]	A random sample of 25/70 radiographs	1	A random sample of 25 of the lateral lumbar radiographs were represented to the examiner and, for each one, the lumbar lordosis was remeasured using methods 1 and 2. The examiner was blind to the first set of measurements.	Standing radfiographs	The lumbar lordosis was measured in degrees using the method of Yochum and Rowe (method 1), utilizing the superior endplate of L1 and the sacral base as landmarks and, in the method of Banks (method 2), using the inferior endplate of L1 and the sacral base as landmarks.	not repoted	The Kappa value using Yochum and Rowe (method 1) was 0.750 and for Banks (method 2) was 0.807, indicating statistically significant intraexaminer agreement in the categorization of the data using each method.	Not reported	H	L	1	0	0	0	0
Wu 2014 [87]	Radiographs of 50 random subjects	Unknown	not described	50 standing lateral digital radiographs.	LL was measured by the Cobb T12 to S1, Pelvic incidence	The interobserver reliability for pelvic incidence (PI) = 0.923 and and for lumbar lordosis (LL) = 0.905.	The interobserver reliability for pelvic incidence (PI) = 0.887 and and for lumbar lordosis (LL) = 0.932.	Not reported	L	H	1	1	0	0	0
Wu 2021 [88]	68 radiographs	3	Three orthopedic surgeons Each observer measured each radiograph twice, with a week's interval between the first and second readings.	68 standard standing whole-spine radiographs were reviewed.	[Lumbar] measurements included pelvic incidence (PI), sacral slope (SS), pelvic tilt (PT), and Lumbar lordosis (LL). Manual measurement on radiographs and SurgimapSpine software ancillary measurement on the computer	not repoted	Intraobserver reproducibility of the manual technique for each observer based on intraclass correlation coefficients (95% confidence interval): Examiner 1: LL = 0.92(0.88-0.95), PI = 0.85(0.77-0.90), SS = 0.89(0.83-0.93), PT = 0.70(0.55-0.80); Examiner 2: LL = 0.90(0.83-0.95), PI = 0.82(0.48-0.92), SS = 0.91(0.86-0.95), PT = 0.70(0.49-0.82); Examiner 3: LL = 0.95(0.89-0.97), PI = 0.86(0.50-0.95), SS = 0.92(0.84-0.95), PT = 0.74(0.57-0.84). Intraobserver reproducibility of the SurgimapSpine technique for each observer based on intraclass correlation coefficients (95% confidence interval): Examiner 1: LL = 0.98(0.97-0.99), PI = 0.87(0.80-0.92), SS = 0.93(0.89-0.96), PT = 0.74(0.62-0.83); Examiner 2: LL = 0.93(0.89-0.96), PI = 0.91(0.89-0.94), SS = 0.92(0.88-0.95), PT = 0.79(0.68-0.87); Examiner 3: LL = 0.95(0.91-0.97), PI = 0.88(0.58-0.95), SS = 0.91(0.85-0.94), PT = 0.77(0.66-0.85).	Not reported	H	L	1	1	1	0	0
Zhang 2022 [89]	30 patients	1	30 pre-operative and post-operativexrays and 3D CT sagittal views of spines. Global [lumbar] measurement included lumbar lordosis (LL).	Xray	Cobb method LL (not detailed)	not repoted	ICC for LL XRay measurement and Pre-OS models (0.955 [0.908-0.978]). ICC for Xray measurement in Post-OS and simulated 3D spine(SS) models (0.896 [0.794-0.949]). Bland-Altman analysis for Xray measurement in Post-OS and SS models (mean bias = -0.2330, limits of agreement = -15.12-10.46).	Not reported	H	L	1	0	0	0	0
Zhou 2021 [90]	62 patients (18 men and 44 women)	1	62 films measured three times by single observer on the five radiographs	Standing flexion and extension radiographs using EOS machine	Slip percentage of L4 spondylolisthesis, and Slip Angle of L5 superior endplate to L4 inferior endplate	not repoted	The slip percentage was measured three times by single observer on these five radiographs using Meyerding's technique (Intra-CC 0.88 [95% CI 0.86 to 0.90]).	Not reported	H	L	0	0	0	1	0
Zhou 2022 [91]	1791 lateral lumbar radiographs	3	three radiologists 3-6 years of experience, respectively) To assess intra-observer reliability, the test set was reannotated by the radiologist (R1) after five weeks.	Lateral lumbar radiographs	L5HA = the vertex of the anterior superior border of L5 vertebra; L5FA = the vertex of the anterior lower border of the L5 vertebra; L5FP = the vertex of the posterior lower border of L5 vertebra; S1HA = the vertex of the anterior superior border of S1 vertebra; S1FA = the vertex of the anterior lower border of S1 vertebra; S1HP = the vertex of the superior posterior border of S1 vertebra; S1FP = the vertex of the posterior lower border of S1 vertebra; LSLA = lumbosacral lordosis angle; LSA = lumbosacral angle; SHA = sacral horizontal angle; SIA = sacral inclination angle.	(%) R1 v R2: 1mm = 26; 2mm = 64; 3mm = 81; 4mm = 89; 5mm = 93; R1 v R3: 1mm = 25; 2mm = 60; 3mm = 79; 4mm = 87; 5mm = 91; R2 v R3: 1mm = 29; 2mm = 66; 3mm = 82; 4mm = 90; 5mm = 93	(%) 1mm = 84; 2mm = 93; 3mm = 97; 4mm = 99; 5mm = 100; LSLA = 0.97 (0.97-0.98); LSA = 0.92 (0.90-0.94); SHA = 0.98 (0.96-0.98); SIA = 0.95 (0.94-0.96)	Not reported	M	M	0	0	1	1	0
Zhu 2015 [92]	100 asymptomatic adults (mean age 37.8 ± 12.4 y); 50 age-matched adult scoliosis adult pts, 31 idiopathic scoliosis & 19 degenerative scoliosis (mean age 41.4 ± 10.8 y)	2	150 films assessed by 2 spine surgeons independently and blindly for inter-observer reliability. After 4 weeks, repeated by 1 surgeon for intra-observer reliability. The time it took to measure was obtained with a stopwatch twice and the average measurement time was analyzed.	Long-cassette standing upright lateral radiographs of spine and pelvis on 14 x 36 in. film at 200-cm tube-to-film distance.	Lumbar lordosis (LL) = L1- S1; LFA defined as the angle between the line connecting the midpoint of the superior plate of S1 to the midpoint of the femoral head axis (Line A) and the line connecting the midpoint of the superior plate of L1 to the midpoint of the femoral head axis (Line B), positive when Line B was in front of Line A; pelvic incidence (PI) pelvic tilt (PT), 6) sacral lope (SS), PI-LL = difference between PI and LL	LL = 0.975 (95% CI 0.931-0.988); PI = 0.920 (95% CI 0.834-0.971); PT = 0.981 (95% CI 0.953-0.992); SS = 0.924 (95% CI 0.883-0.967); PI-LL = 0.927 (95% CI 0.897-0.971); LFA = 0.937 (95% CI 0.858-0.974); TK = thoracic kyphosis, LL = lumbar lordosis, PI = pelvic incidence, PT = pelvic tilt, SS = sacral slope, PI-LL = pelvic incidence minus lumbar lordosis, LFA = lumbo-femoral angle	LL = 0.982 (95% CI 0.976-0.996); PI = 0.897 (95% CI 0.858-0.912); PT = 0.892 (95% CI 0.775-0.968); SS = 0.853 (95% CI 0.753-0.929); PI-LL = 0.924 (95% CI 0.873-0.952); LFA = 0.961 (95% CI 0.954-0.981)	Not reported	M	M	1	1	1	0	0
**Totals**											43	22	19	19	4
