# Supplementary material for: Reliability of the Biomechanical Assessment of the Sagittal Lumbar Spine and Pelvis on Radiographs Used in Clinical Practice: A Systematic Review of the Literature
Source: J Clin Med. 2024 Aug 8;13(16):4650. doi: 10.3390/jcm13164650 (PMC11355792; doi:10.3390/jcm13164650)
Supplement: Supplementary file 1 [file jcm-13-04650-s001.zip › Table S2-Intra-examiner-subgroups-WORD-refsNumbered.pdf]

Table S2. Intra-examiner reliability studies grouped by analysis method and study quality. ICC: intraclass correlation coefficient; SEM: standard error of measurement; MAD: mean absolute value of observer differences; N: number; Obs.: Observer; X: non-existent; SD: standard deviation; LOA: limit of agreement; CI: confidence interval; PI: pelvic incidence; PT: pelvic tilt; PA: pelvic angle; Avg: average.

Mensuration Method	Low Quality	Intra-Examiner Reliability Statistics	Moderate Quality	Intra-Examiner Reliability Statistics	High Quality	Intra-Examiner Reliability Statistics	Intra-Examiner Reliability Quality	SEM, MAD
**Harrison Posterior Tangent Method (ARA L1–L5)**	N = 2 [57, 71]	ICC = 0.85 [57]<br>Pooled all methods Kappa obs. 1 = 0.226, obs. 2 = 0.542 [71]	N = 1 [71]	ICC Obs. 1 = 0.991, Obs 2 = 0.989 [71]	N = 4 [46,49,82,83]	ICC = 0.99 [46]<br>ICC = 0.96 [49]<br>ICC = 0.98 [82]<br>ICC = 0.98 [83]	Excellent [46,49,57,72,82,83]	MAD = 3.2 [57]<br>MAD = 1.8 [46]<br>MAD = 3.23 [49]
**TRALL Method**	N = 2 [57,71]	ICC = .77 [57]<br>Pooled all methods Kappa obs. 1 = 0.226, obs. 2 = 0.542 [71]	N = 0	X	N = 2 [46,49]	ICC = 1.00 [46]<br>ICC = 0.93 [49]	Excellent [46,49]	MAD = 0.8 [46]<br>MAD = 3.0 [49]
**Cobb Method (T12–L5)**	N = 0	X	N = 0	X	N = 1 [68]	ICC = 0.83, 0.85, 0.96 [68]	Good–Excellent [68]	Not Reported
**Cobb Method (T12–S1)**	N = 0	X	N = 2 [66,74]	ICC = 0.77 [66]<br>ICC = 0.78 [74]	N = 4 [46,68,83,87]	ICC = 0.99 [46]<br>ICC = 0.91, 0.85, 0.95 [68]<br>ICC = 0.88–0.95 [83]<br>ICC = 0.932 [87]	Good–Excellent [68]<br>Excellent [46,66,74,83,87]	MAD = 1.6 [46]
**Cobb Method (L1–L5)**	N = 1 [32]	ICC = 0.83 [32]	N = 3 [33,38,72]	ICC = 0.94–0.99 [33]<br>ICC = 0.983–0.968 72	N = 4 [31,49,56,68]	ICC = 0.95 [49]<br>ICC = 0.935 [56]<br>ICC = 0.89, 0.83, 0.94 [68]	Excellent [32,33,49,56,68,72]	SEM < 1 deg [32]<br>MAD = 2.0 [38]<br>MAD = 2.53 [49]

				Pearson =0.918 – 0.972 [38]		Bland-Altman LOA 95% CI = 47–59 deg [31]	Good [31]<br>Very High Correlation [38]	
**Cobb Method (L1–S1)**	N = 3 [40,71,86]	Kappa pooled results: Obs. 1 = 0.226, Obs. 2 = 0.542 [71]<br>Kappa method 1 = 0.750, method 2 = 0.807 [86]	N = 7 [30,34,38,55, 58,66,92]	ICC = 0.966–0.992 [34]<br>ICC = 0.983, 0.986, 0.986 [55]<br>ICC = Method 1 = 0.98, Method 2 = 0.96 [58]<br>ICC = 0.77 [66]<br>ICC = 0.982 [92]	N = 4 [31,49,56,68]	ICC = 0.97 [49]<br>ICC = 0.89, 0.83, 0.94 [68]	Good–Excellent [68]<br>Excellent [34,49,55,58,66,92]	Limits of agreement, mean Difference plus or minus 2 SDs, are 6.79° and −5.69° [40]<br>MAD = 2.02 deg [38]
		Bland Altman LOA: Obs. 1 = 0.1° ± 2.3° (t = -0.1951; df = 19; P = 0.8474); Obs. 2 = 0.5° ± 2.2° [40]		Fleiss k = 0.627 [30]		Bland-Altman LOA 95% CI = 47–59 deg [31]	Good [30,31]	
				Pearson = 0.894–0.973 [38]			Very High Correlation [38]	
**Cobb Method (undefined)**	N = 2 [88,89]	ICC = 0.92, 0.90, 0.95 [88]<br>ICC = 0.955, 0.896 [89]	N = 4 [41,54,75,76]	ICC = 0.840 [75]<br>ICC = 0.92 [76]	N = 1 [78]	ICC = 0.96, 0.98, 0.99 [78]	Excellent [54,75,76,78,88,89]	Not Reported
		Bland Altman LOA: mean bias = -0.2330, limits of agreement = -15.12–10.46 [89]		Kappa: 0.47–0.72 [41]			Fair to Moderate [41]	
				Pearson: 0.86 [54]				
**Lumbar Lordosis (Other, e.g., Centroid, Pelvic-radius technique, etc.)**	N = 3 [57,71,86]	ICC =0.90–0.97 [57]	N = 4 [38,50,51,80]	ICC = 0.96 and 0.99 [80]	N = 1 [49]	ICC = 0.85 [49]	Excellent [38,49–51,80]	Not Reported
		Kappa: pooled results: Obs. 1 = 0.226, Obs. 2 = 0.542 [71]		Pearson = 0.904–0.982 [38]<br>Pearson = Obs. 1: 0.95–0.99,			Weak [71]	

		Kappa method 1 = 0.750, method 2 = 0.807 [86]		Obs. 2: 0.87–0.99 [51] Pearson = 0.94–0.98 [50]				
**Sacral Slope**	N = 1 [88]	ICC: Method 1 = 0.89, 0.91, 0.92, Method 2 = 0.93, 0.92, 0.91 [88]	N = 8 [34,41,44,58, 62,75,91,92]	ICC = 0.944–0.983 [34] ICC = 0.64 [41] ICC = 0.92, 0.95 [44] ICC = 0.92 [58] ICC = 0.958–0.98 [62] ICC = 0.840 [75] ICC = 0.95 [91] ICC = 0.853 [92]	N = 6 [39,46,56,78, 82,83]	ICC Film 1 = 0.869–0.970, Film 2 = 0.899–0.972 [39] ICC = 0.99 [46] ICC = 0.943 [56] ICC = 0.95, 0.98, 0.99 [78] ICC = 0.91–0.97 [82] ICC = 0.83–0.94 [83]	Good [41] Excellent [34,44,39,46, 56,58,62, 75,78,82,83, 88,91,92]	MAD = 1.2 deg [46]
**Pelvic Incidence/Pelvic Tilt**	N = 0	X	N = 10 [30,41, 50,54,55,58, 62,75,76,92]	ICC: PI = 0.69, PT = 0.60 [41] ICC: PA = 0.96–0.99 [50] ICC: PI = 0.78, PT = 0.86 [54] ICC: PI = 0.984, 0.990, 0.966, PT = 0.989, 0.997, 0.958 [55] ICC: Method 1: PI = 0.98, PT = 0.97, Method 2: PI = 0.95, PT 0.93 [58] ICC: PI = 0.958–0.987, PT = 0.978–0.999 [62] ICC: PI = 0.840, PT = 0.821 [75]	N = 4 [39,56,78,87]	ICC: Film 1: PI = 0.882–0.982; Fim 2: PI = 0.846–0.975; Film 1: PT = 0.959–0.984; Film 2: PT = 0.822–0.996 [39] ICC: PI = 0.956, PT = 0.978 [56] ICC: Obs. 1: PI = 0.97, PT = 0.99; Obs. 2: PI = 0.98, PT = 1.00; Obs. 3: PI = 0.99, PT = 1.00 [78] ICC: PI = 0.887 [87]	Excellent [39,50,54–56,58,62,75,76, 78,87,92]	Not Reported

				ICC: PI = 0.94, 0.97, PT = 0.93, 0.97 [76]<br>ICC: PI = 0.987, PT = 0.892 [92]				
				Pearson: PI = 0.65, PT = 0.55 [41]			High correlation [41]	
				Fleiss k: PT = 0.633, PI–LL = 0.627 [30]			Substantial [30]	
**Harrison Posterior Tangent Method (RRA L1–L5)**	N = 0	X	N = 0	X	N = 3 [46,82,83]	ICC = 0.88–0.97 [46]<br>ICC = 0.78–0.94 [82]<br>ICC = 0.55–0.97 [83]	Excellent [46,82,83]	Not Reported
**Segmental Cobb**	N = 0	X	N = 3 [36,42,61]	ICC = 0.96–0.99 [42]<br>ICC: Flexion: L2/3 = 0.959, L3/4 = 0.981, L4/5 = 0.997; Extension: L2/3 = 0.959, L3/4 = 0.920, L4/5 = 0.993 [61]	N = 4 [29,43,65,79]	Avg. ICC = 0.653 [29]<br>ICC = 0.81–0.96 [43]<br>ICC = Method 1 = 0.708–0.806, Method 2 = 0.962–0.990 [65]	Moderate [29] Excellent [42,43,61]	Not Reported
							Very High to High Correlation [36]	Not Reported
				Pearson = Obs. 1: 0.902, t test = 0.17° (0.617); Obs. 2: 0.782, t test = 0.04° (0.932) [36]		L3–L4 level 1.4° (SD 1.3°), at L4–L5 1.5° (SD 1.3°) and at L5–S1 1.6° (SD 1.6) [79]	Low to Excellent [65]	Not Reported

**Global Sagittal Translation (T12–S1)**	N = 0	X	N = 0	X	N = 3 [46,82,83]	ICC = 1.00 [46] ICC = 0.97–0.99 [82] 0.98–1.00 [83]	Excellent [46,82,83]	MAD = 0.6 mm [46]
**Segmental Translation**	N = 1 [90]	ICC = 0.88 [90]	N = 5 [60,67,80, 81,91]	ICC = 0.69–0.87 [60] ICC = 0.84 [80] ICC = 0.94 [81]	N = 5 [43,60,65,80,81]	ICC = 0.84–0.99 [43] ICC: Method 1 = 0.083–0.671, Method 2 = 0.234–0.849 [65]	Low to Excellent [65] Moderate to Excellent [60] Excellent [43,80,81]	SEM = 0.4–0.7 mm [80]
				Pearson: L5 retrolisthesis = 0.90 [67]		L3–L4 level 0.7 mm (SD 0.7 mm), at L4–L5 0.7 mm (SD 0.7 mm) and at the L5–S1 level 0.6 mm (SD 0.8 mm) [79]	Excellent [67]	
				(% repeat analysis) 1 mm = 84%; 2 mm = 93%; 3 mm = 97%; 4 mm = 99%; 5 mm = 100% [91]		Kappa = 0.83 (range, 0.77–0.89) [53]		
