# Supplementary material for: Reliability of the Biomechanical Assessment of the Sagittal Lumbar Spine and Pelvis on Radiographs Used in Clinical Practice: A Systematic Review of the Literature
Source: J Clin Med. 2024 Aug 8;13(16):4650. doi: 10.3390/jcm13164650 (PMC11355792; doi:10.3390/jcm13164650)
Supplement: Supplementary file 1 [file jcm-13-04650-s001.zip › Table S3-Inter-examiner-subgroups-WORD-refsNumbered.pdf]

Table S3. Inter-examiner reliability studies grouped by analysis method and study quality. ICC: interclass correlation coefficient; SEM: standard error of measurement; MAD: mean absolute value of observer differences; N: number; Obs.: Observer; X: non-existent; SD: standard deviation; LOA: limit of agreement; CI: confidence interval; PI: pelvic incidence; PT: pelvic tilt; PA: pelvic angle; PR: pelvic radius; Avg: average.

Mensuration Method	Low Quality	Inter-Examiner Reliability Statistics	Moderate Quality	Inter-Examiner Reliability Statistics	High Quality	Inter-Examiner Reliability Statistics	Inter-Examiner Reliability Quality	SEM, MAD
**Harrison Posterior Tangent Method (ARA L1–L5)**	N = 2 [57,71]	ICC = 0.90 [57]<br>Pooled kappa = 0.36, 0.16 [71]	N = 1 [72]	ICC = 0.985 [72]	N = 4 [46,49,82,83]	ICC = 0.98 [46]<br>ICC = 0.91 [49]<br>ICC = 0.98 [82]<br>ICC = 0.96 [83]	Excellent [46,57,72,82,83]<br>no agreement to slight agreement [71]	SEM = 0.8–1.0° [46]<br>MAD = 5.6° [57]
**TRALL Method**	N = 2 [57,71]	ICC = 0.83 [57]<br>Pooled kappa = 0.36, 0.16 [71]	N = 0	X	N = 2 [46,49]	ICC = 0.99 [46,49]	Good [57,49]<br>Excellent [46]<br>no agreement to slight agreement [71]	SEM = 0.80–1.0° [46], SEM = 3.27° [49]<br>MAD = 6.4° [57]
**Cobb Method (T12–L5)**	N = 0	X	N = 0	X	N = 1 [68]	ICC = 0.87	Good [68]	Not Reported
**Cobb Method (T12–S1)**	N = 1 [37]	Pearson = 0.9687–0.9952 [37]	N = 2 [66,74]	ICC = 0.97 [66],<br>ICC = 0.93 [74]	N = 4 [46,68,83,87]	ICC = 0.98 [46]<br>ICC = 0.85 [68]<br>ICC = 0.88 [83]<br>ICC = 0.905 [87]	Good [68,83]<br>Excellent [46,66,74,87]<br>Excellent [37]	Not Reported
**Cobb Method (L1–L5)**	N = 2 [32,70]	ICC = 0.94 [32],<br>0.90 [70]	N = 4 [33,38,47,72]	ICC = 0.96 (Digital) [33]<br>ICC = 0.94 (Plain Film) [33]<br>ICC = 0.826 [38]<br>ICC = 0.98 [47]<br>ICC = 0.952 [72]	N = 5 [39,46,49,59, 68]	ICC: whole spine lateral = 0.956–0.975, lateral lumbar = 0.948–0.964 [39],<br>ICC = 0.98 [46],<br>ICC = 0.91 [49],<br>ICC = 0.765 [59],<br>ICC = 0.91 [68]	Good [38,59]<br>Excellent [32,33,39,46,47,49,68, 70,72]	Not Reported

**Cobb Method (L1–S1)**	N = 4 [40,63, 70,71]	ICC = 0.85 [70]	N = 8 [30,34,38,48, 55,58,66,92]	ICC = 0.944 [34]<br>ICC = 0.906 [48]<br>ICC = 0.980–0.993 with smartphone [55]<br>ICC = 0.865–0.974 with protractor [55]<br>ICC = 0.93 [58]<br>ICC = 0.97 [66]<br>ICC = 0.975 [92]	N = 4 [31,49,56,68]	ICC = 0.90 [49]<br>ICC = 0.872 [56]<br>ICC = 0.83 [68]	Good [56,68]<br>Excellent [34,48,49,55,58,66,70, 92]	Not Reported
		Pooled kappa = 0.36, 0.16 [71]		Pearson = 0.784 [38]			Strong Correlation [38]	
		Bland Altman LOA: examiner1 = 4.48° and −4.68°, examiner 2 = 6.79° and −5.69° [40]		Fleiss k coefficients: (round 1 / round 2) PI–LL = [0.554 / 0.386, 0.826] [30]		Bland-Altman LOA 95% CI = 47°–59° [31]	No agreement to slight agreement [71]<br>Substantial agreement [30]	
		SD = 12.8° [63]					good agreement (Andreason)	
**Cobb Method (undefined)**	N = 1 [85]	ICC = 0.90 [85]	N = 4 [41,54,75,76]	ICC: manual method = 0.57 [41]<br>ICC: computerized = 0.90 [41]<br>ICC = 0.67 [54]<br>ICC = 0.703 [75]<br>ICC = 0.96 [76]	N = 2 [45,78]	ICC = 0.961 [45]<br>ICC = 0.96 [78]	Moderate [41–manual method, 54,75]<br>Excellent [41–computerized method, 85,76,45,78]	Not Reported
**Lumbar Lordosis (Other, e.g., Centroid, Pelvic-radius**	N = 3 [37,57, 71]	ICC = 0.86–0.96 [57]	N = 3 [38,51,50]		N = 1 [49]	ICC = 0.85 [49]	Good [49]<br>Good-to-Excellent [57]	Not Reported
		Pearson = 0.9687–0.9952 [37]		Pearson = 0.903 [38]			Moderate-to-Excellent [51]<br>Excellent [37,38,50]	

**technique, etc.)**				Pearson = 0.84–0.97 [51]<br>Pearson = 0.94–0.98 [50]				
		kappa = 0.36, 0.16 [71]					no agreement to slight agreement [71]	
**Sacral Slope**	N = 2 [63,70]	ICC = 0.96 [70]	N = 10 [34,41,44,48, 58,62,64,75, 91,92]	ICC = 0.990 [34]<br>ICC = 0.86 [44]<br>ICC = 0.872 [48]<br>ICC = 0.89 [58]<br>ICC = 0953–0.97 [62]<br>ICC = 0.866 [75]<br>ICC = 0.98 [91]<br>ICC = 0.924 [92]	N = 6 [39,46,56,78, 82,83]	ICC = 0.911, 0.939, 0.916 [39]<br>ICC = 0.99 [46]<br>ICC = 0.877 [56]<br>ICC = 0.96 [78]<br>ICC = 0.95 [82]<br>ICC = 0.82 [83]	Good [44,48,56,58,75,83]<br>Excellent [34,39,46,62,70,78,82, 91,92]	Mean Absolute Error = 1.62° [91], MAD = 1.2° [46], SEM = 1.73° [82]
		SD = 5.7° [63]		Pearson = 0.71 [41]<br>Pearson = 0.92 [64]			Strong Correlation [41,64]	
**Pelvic Incidence/Pelvic Tilt**	N = 3 [37,52, 70]	ICC: PI = 0.88, PT = 0.89 [52],<br>ICC: PI = 0.97 [70]	N = 12 [30,41,48,50, 54,55,58,62, 64,75,76,92]	ICC (Manual): PI = 0.41 (0.36–0.45), PT = 0.42 (0.37–0.46) [41]<br>ICC (Computerized): PI = 0.98 (0.98–0.99), PT= 0.99 (0.99–1.00) [41]<br>ICC PI = 0.913, PT = 0.970 [48]<br>ICC: PI = 0.42, PT = 0.63 [54]<br>ICC: PI (smartphone) = 0.970–0.975, PT (smartphone) = 0.874–0.969; PI	N = 4 [39,56,78,87]	ICC: PT on whole-spine radiographs = 0.980–0.986, PT on lateral lumbar radiographs = 0.945–0.970 [39]<br>ICC: PI = 0.909, PT = 0.978 [56]<br>PI = 0.98, PT = 0.99 [78]<br>ICC: PI = 0.923 [87]	Poor [41–manual method]<br>Poor-to-Moderate [54] Moderate [52,75]<br>Moderate-to-Excellent [55,58]<br>Excellent [41–computerized method, 48,62,64,70,76,92, 39,56,78,87]	Not Reported

				(protractor) = 0.86–0.941, PT (protractor) = 0.807–0.959 [55]<br>ICC (PACS): PI = 0.86, PT = 0.90; (APP): PI = 0.84, PT = 0.93 [58]<br>ICC: PI = 0.951–0.977, PT = 0.966–0.988 [62]<br>ICC: PI = 0.96, 0.95, PT = 0.93, 0.94 [64]<br>ICC: PI = 0.653, PT = 0.674 [75]<br>ICC: PI = 0.97, PT = 0.96 [76]<br>ICC: PI = 0.920, PT = 0.981 [92]				
		Pearson = 0.9687–0.9952 with P value < 0.0001 [37]		Pearson: PR and PA 0.94–0.98 [50], PI = 0.96, 0.93, PT = 0.96, 0.95 [64]			Excellent [37,50,64]	
				Fleiss k coefficients: (round 1/round 2) PT = 0.714, PI–LL = [0.554/0.386, 0.826] [30]			Moderate Agreement [30]	
**Harrison Posterior Tangent Method (RRA L1–L5)**	N = 0	X	N = 0	X	N = 3 [46,82,83]	ICC = 0.83–0.96 [46]; 0.68–0.98 [82]; 0.65–0.89 [83]	Good-to-Excellent [46], Moderate-to-Excellent [82], Moderate-to-Good [83]	Not Reported
**Segmental Cobb**	N = 0	X	N = 4 [36,42,61,73]	ICC = 0.94–0.99 [42]	N = 3 [29,65,79]	ICC = 0.0902 [29]	Excellent [29,42,61,65]	Not Reported

				ICC: Flexion: L2/3 = 0.912, L3/4 = 0.975, L4/5 = 0.967; Extension: L2/3 = 0.761, L3/4 = 0.737, L4/5 = 0.988 [61]		ICC manual method ranged from 0.484 (L1–L2) to 0.780 (L3–L4), computerized method ranged from 0.912 (L5–S1) to 0.962 (L4–L5) [65]		
				Pearson = 0.921 (0.929–0.913) [36]			Excellent [36]	
				Proportion of Agreement = 0.93, 0.99, kappa coefficient = 0.35, 0.57, Agreement coefficient 0.91, 0.86 [73]		mean segmental angular variation (Reader 1 vs. 2; 1 vs. 3 and 2 vs. 3) at the L3–L4 level was 1.9° (SD 1.4°), at L4–L5 2.3° (SD 1.9°) and at the L5–S1 level 2.6° (SD 2.3°) [79]	High reproducibility and agreement [73]	
**Global Sagittal Translation (T12–S1)**	N = 0	X	N = 0	X	N = 3 [46,82,83]	ICC = 1.00 [46] 0.97–0.99 [82] 0.98–1.00 [83]	Excellent [46, 82, 83]	MAD = 0.6 mm [46]
**Segmental Translation**	N = 0	X	N = 5 [60,67,73,81, 91]	ICC = 0.59 for Tallard method (L5 spondylolisthesis), to 0.85 for Boxall method (L5	N = 4 [35,53,65,79]	ICC = 0.64–0.92 [35] ICC: AP translation: computer assisted = 0.862, manual = 0.151 [65]	Moderate-to-Excellent [35] Poor for manual method [65] Excellent for computer method [65]	Not reported

				spondylolisthesis) [60]				
				Pearson r: L5 retrolisthesis = 0.74, 0.79, 0.83 [67]		mean variation: L3–L4 = 1.3 mm (SD 1.1 mm), L4–L5 = 1.4 mm (SD 1.2 mm), L5–S1 = 1.3 mm (SD 1.4 mm) [79]		
				Proportion of Agreement = 0.90–0.94, kappa coefficient = -0.03–0.57, Agreement coefficient = 0.86–0.94 [73]<br>Kappa = 0.78 for Meyerding classification [81]<br>% agreement between 3 examiners within: 1 mm = 25, 26 and 29%, within 2 mm = 60, 64 and 66%, within 3 mm = 79, 81, 82%, within 4 mm = 87, 89 and 90%, within 5 mm = 91, 93 and 93% [91]		Kappa = 0.82 [53]	High reproducibility and agreement [73,81,91]<br>Near perfect agreement [53]	
